# ELGCot3D: a lightweight 3D cotton point cloud segmentation model based on EdgeConv-Local Attention-GCN and semantic feature enhancement

**DOI:** 10.3389/fpls.2026.1765604

**Published:** 2026-02-06

**Authors:** Hao Qiu, Xiaoyan Meng, Yongke Li, Yunjie Zhao, Xiaoyu Li, Shuai Yin, Haoyuan Niu

**Affiliations:** 1 School of Computer and Information Engineering, Xinjiang Agricultural University, Urumqi, China; 2 Ministry of Education Engineering Research Center for Intelligent Agriculture, Urumqi, China; 3 Xinjiang Agricultural Informatization Engineering, Technology Research Center, Urumqi, China

**Keywords:** 3D point cloud, cotton organ segmentation, ELG3D, ELGCot3D, feature enhancement, lightweight

## Abstract

Efficient and non-destructive cotton organ extraction is crucial for automatic cotton phenotyping. However, limited by leaf occlusion, large model parameters, and inefficient manual observation, it fails to meet current high-throughput phenotyping demands. To address these challenges, this paper propose ELGCot3D, a lightweight 3D point cloud-based cotton organ segmentation method, enabling high-precision segmentation in resource-constrained environments. First, a new module called ELG3D replaces traditional Set Abstraction structures, enhancing local cotton data learning capability via multi-mechanism feature fusion and boosting segmentation accuracy. Second, a cotton-specific feature enhancement module is proposed to secondary optimize the features output from the Feature Propagation layer. This module significantly increases feature discriminability while substantially reducing redundant and high consumption network layers, achieving a balance between performance and efficiency. Finally, a cotton point cloud-adapted training strategy improves model training stability and prediction accuracy. Experimental results on the Crops3D dataset show ELGCot3D achieves 76.7% mIoU and 86.1% OA for cotton segmentation, Meanwhile, the number of parameters and computational complexity are reduced by 50.1% and 50.7%, respectively, demonstrating the model’s lightweight characteristics. Notably, it performs well in segmenting other Crops3D crops and exhibits strong generalization on the other cotton point cloud datasets. The proposed method offers a reliable approach for cotton phenotyping and precision agriculture. Future work will extend its high-throughput extraction capability for individual plant organs in large cotton fields, providing breeders with accurate data to support efficient breeding and new variety development.

## 1 Introduction

Cotton, as a globally important cash crop (Zhu et al., 2025), its fiber products support the core supply chain of the textile industry, while its seed oil (Younas et al., 2025) and by-products also have irreplaceable value in fields such as food (Lv et al., 2025) and chemical engineering (Yan et al., 2025). According to statistics from the Food and Agriculture Organization of the United Nations (FAO) (OECD/FAO, 2024), the global annual planting area of cotton exceeds 30 million hectares, directly related to the livelihood of hundreds of millions of people and the agricultural economic structure of dozens of countries. In modern agriculture, the core demand of the cotton industry has shifted from mere yield increase to a production model emphasizing both high-quality and efficient precision management (Xiao et al., 2024). The realization of this goal is highly dependent on the accurate quantification of cotton phenotypic traits. Studying cotton phenotypes not only enables a more precise understanding of plant physiological status, adaptability, and environmental responses (Hao et al., 2024) but also serves as a key basis for selecting high-yield and high-quality genotypes in crop breeding (Liu et al., 2025b), as well as core data support for formulating precision cultivation measures such as field water and fertilizer regulation (Jia et al., 2025) and pest early warning (Li et al., 2024).

Traditional crop phenotyping relies on manual observation and counting, which is time-consuming, labor-intensive, and susceptible to the observer’s subjective experience, making it difficult to meet the needs of large-scale breeding trials or dynamic field monitoring (Gupta et al., 2005). With the development of relevant demand-driven technologies, current crop phenotyping analysis technologies focus on 2D images and 3D point clouds (Gené-Mola et al., 2020; Sun et al., 2020; Zhang et al., 2023a). Among them, 2D images emerged as the core of early research and applications for convenient data acquisition. The technical route has evolved from traditional algorithms like threshold segmentation (Fu et al., 2019) and edge detection (Wang et al., 2018) to breakthroughs driven by deep learning. For instance, Sadeghi-Tehran et al. (Sadeghi-Tehran et al., 2019) realized wheat ear semantic segmentation by combining simple linear iterative clustering with CNN, and Yi et al. (Yi et al., 2023) modified the UNet network for accurate segmentation of birch stems and leaves. However, 2D images have inherent limitations: lack of 3D spatial information, easy key information loss caused by organ occlusion, and image quality interference from light and shadows. These issues are particularly prominent in complex field or fine phenotyping scenarios (Wei et al., 2025). With the development of computer vision and 3D perception technologies, 3D point cloud technology has gradually become a core tool in phenotyping research (Gu et al., 2024). Compared with 2D images that only capture planar texture information, it can fully reconstruct the three-dimensional morphology of cotton plants, accurately restore the spatial positional relationship between organs, effectively solve the occlusion problem, and exhibit stronger robustness to environmental interference, providing a more reliable data source for organ-level segmentation and parameter extraction (Minervini et al., 2015; Gibbs et al., 2016). This advantage has been fully verified in field crop point cloud segmentation research. For instance, Shen et al. (Shen et al., 2024) realized organ segmentation and phenotypic trait extraction of cotton seedling point clouds based on a 3D lightweight network. Liu et al (Liu et al., 2025a). proposed the TPointNetPlus model integrated with a Transformer attention module to enhance the feature discrimination ability of occluded regions. Lin et al. (Lin et al., 2022) employed 3D laser point cloud technology to develop a 3D information interpretation model for organs using field maize point cloud data. Yao et al. (Yao et al., 2025) leveraged unmanned aerial vehicle (UAV) 3D point cloud coupled with the improved PointNet++ technology to achieve automated determination of tobacco phenotypic traits, and Dong et al. (Dong et al., 2025) combined PointNeXt with the Quickshift++ algorithm to realize instance segmentation of multiple crop organs.

Despite the significant advantages of 3D point cloud technology (Qi et al., 2017b; Zhao et al., 2021), research on cotton still faces multiple challenges. Cotton plants have multiple leaf layers, dense interlaced branches and leaves, and similar morphologies between bolls and leaves, leading to severe organ occlusion. Traditional point cloud segmentation models struggle to effectively distinguish the boundaries of overlapping organs. Beyond these technical challenges inherent in cotton point cloud analysis itself, practical application scenarios pose additional hurdles. Implementing cotton phenotyping analysis in field deployment faces multiple challenges. A major algorithmic bottleneck lies in the fact that existing high-precision 3D segmentation models [e.g., PointMLP (Ma et al., 2022), CurveNet (Muzahid et al., 2020)] are often parameter-heavy and computationally complex, making it difficult to meet the requirements for low-power, real-time inference on resource-constrained platforms like drones. Therefore, improving computational efficiency is the primary objective of this study. Furthermore, transitioning from the lab to field application involves overcoming more front-end challenges, such as robustly isolating individual plants from dense crop point clouds (i.e., instance segmentation). Currently, this study focuses on achieving high-precision, lightweight organ segmentation on already isolated individual plant point clouds, which is a crucial core component for building a complete field-based phenotyping analysis pipeline. Beyond these practical implementation challenges, a deeper underlying factor that exacerbates such difficulties lies in the difference in core goals between crop phenotypic segmentation and general point cloud segmentation. The former needs to adapt to the structural characteristics of crops such as dense overlapping leaves and similar organ morphologies, as well as the deployment constraints of agricultural scenarios. Its core difficulties focus on fine organ-level separation and field data noise interference (Zhu and Yu, 2025), while the latter only pursues universal accuracy. Although general point cloud segmentation models perform excellently in conventional 3D object recognition tasks, they are difficult to directly adapt to crop scenarios. Existing crop-specific research mainly focuses on crop adaptation optimization and lightweight deployment of general architectures, and there are still obvious shortcomings in dedicated solutions (Chen et al., 2025). Turgut et al. (Turgut et al., 2022) adopted the deep learning network RoseSegNet based on 3D point clouds to successfully segment organs such as leaves, stems, and flowers from 3D point cloud data of rose bushes. Song et al. (Song et al., 2025) specifically realized cotton organ segmentation, including stems, petioles, and leaves, through the deep learning network CotSegNet. Ghahremani et al. (Ghahremani et al., 2021) utilized the deep learning model Pattern-Net to realize semantic segmentation of wheat ears and non-ear wheat organs for 3D point cloud data of wheat. Yang et al. (Yang et al., 2025) employed the deep learning model PlaneSegNet to achieve accurate segmentation of plant and non-plant targets, and validated its efficacy on the Crop3D dataset (Zhu et al., 2024). Yet this model is plagued by an excessively large number of parameters. Notably, this drawback is not exclusive to PlaneSegNet. In fact, it epitomizes a prevalent dilemma across similar deep learning-based 3D point cloud segmentation methods. Although such approaches deliver outstanding segmentation accuracy, they impose stringent demands on hardware resources, and their robustness in complex field environments remains to be enhanced. Addressing these issues is intrinsically tied to the core technical bottlenecks of 3D point cloud-based crop semantic segmentation.

Modern 3D point cloud crop semantic segmentation is mainly based on deep learning (Zhang et al., 2019), with the core challenge lying in handling the disorder, sparsity, and complex spatial correlations of point clouds (Zhang et al., 2023b). The early representative work PointNet (Qi et al., 2017a) pioneered the use of symmetric functions to address the disorder of point clouds, utilizing MLP (Taud and Mas, 2017) to directly map individual point features for end-to-end classification and segmentation. However, it can only capture global features and lacks the ability to model local neighborhood structures, making it difficult to handle occluded or dense point cloud scenarios. To overcome this limitation, progressive feature aggregation of “downsampling-local grouping-feature extraction” is achieved through the Set Abstraction (SA) module, which significantly improves the ability to capture local geometric structures. In the SA module, Farthest Point Sampling selects key center points (Li et al., 2022), grouping operations construct local neighborhoods, and then a mini-PointNet extracts neighborhood features, enabling the model to gradually learn hierarchical features from local to global. This architecture has become the foundation for subsequent point cloud segmentation models, but it still suffers from insufficient local feature discrimination when processing highly overlapping or morphologically similar objects. With in-depth research, researchers have optimized point cloud feature extraction mechanisms from various perspectives. Graph convolution (Bhatti et al., 2023) based models [e.g., Point-GNN (Shi and Rajkumar, 2020), DGCNN (Phan et al., 2018)] regard point clouds as topological graphs, model point-to-point relationships through adjacency matrices, and transmit spatial correlation information using graph convolution operators, enhancing adaptability to non-Euclidean structures. CurveNet (Muzahid et al., 2020) aggregates and synthesizes curve sequences in point clouds through a guided walking strategy, and aggregates curve features back to point features to enhance geometric description ability, significantly improving the recognition accuracy of complex surfaces and fine components. PointCNN (Li et al., 2018) is a deep learning algorithm that directly processes unstructured point clouds. It learns local neighborhood features of each point through X-Conv operators, enabling core tasks such as point cloud classification and segmentation without converting point clouds into structured forms such as voxels or images. PointMLP abandons complex geometric extractors and relies on a residual MLP architecture and geometric affine modules to dynamically adjust local features, achieving efficient local feature aggregation.

However, existing architectures still face unique challenges in practical agricultural scenarios (Saeed and Li, 2021; Guo et al., 2023). On the one hand, the dense occlusion and morphological convergence of crop organs place higher requirements on the model’s local feature discrimination ability. On the other hand, the hardware resource constraints of field deployment further strengthen the demand for model lightweighting. Most existing models either focus on accuracy improvement, leading to a surge in parameter size and computational cost, or simplify the network structure to pursue inference efficiency, thereby sacrificing segmentation performance, making it difficult to achieve an effective balance between accuracy and efficiency. At the same time, relevant phenotypic research on structurally complex crops such as cotton remains scarce. To address the core issues of complex and indistinguishable plant structures (Shen et al., 2024) and large model parameter sizes in current intelligent cotton organ extraction, this paper proposes ELGCot3D, a lightweight 3D point cloud cotton organ segmentation method. Unlike mainstream models such as the traditional PointNet++ and PointMLP, which are typically plagued by excessively large parameter volumes and limited to general-purpose segmentation tasks, this method takes the synergistic optimization of high precision and lightweighting as its core design objective. It thereby enables efficient, non-destructive extraction of cotton organ point clouds even in resource-constrained environments, through the deployment of improved network modules and tailored training strategies. The main work and contributions of this paper are as follows:

1. The traditional Set Abstraction module is reconstructed using the EdgeConv-Local Attention-GCN Hybrid 3D Cotton Phenotype Segmentation Module (ELG3D), and EdgeConv, local attention mechanism, and graph convolution are adopted to further enhance the ability to capture local features of overlapping cotton organs and alleviate the problem of low segmentation accuracy in occluded regions.
2. A feature enhancement module suitable for cotton plant structures is designed to perform secondary optimization on the high-level features output by the Feature Propagation layer. While improving feature discriminability, it achieves a balance between model performance and computational efficiency by streamlining redundant network layers and introducing efficient convolution operators.
3. An innovative training strategy adapted to the distribution of cotton point cloud data is proposed. By combining the proposed MuSGD hybrid optimizer, cosine annealing learning rate scheduler, and targeted data augmentation, it significantly enhances the training effectiveness of the lightweight model, improves the model’s adaptability to cotton phenotypes with different morphological structures, and further stabilizes the segmentation performance.

## 2 Methods

### 2.1 ELGCot3D architecture

**Figure 1:**
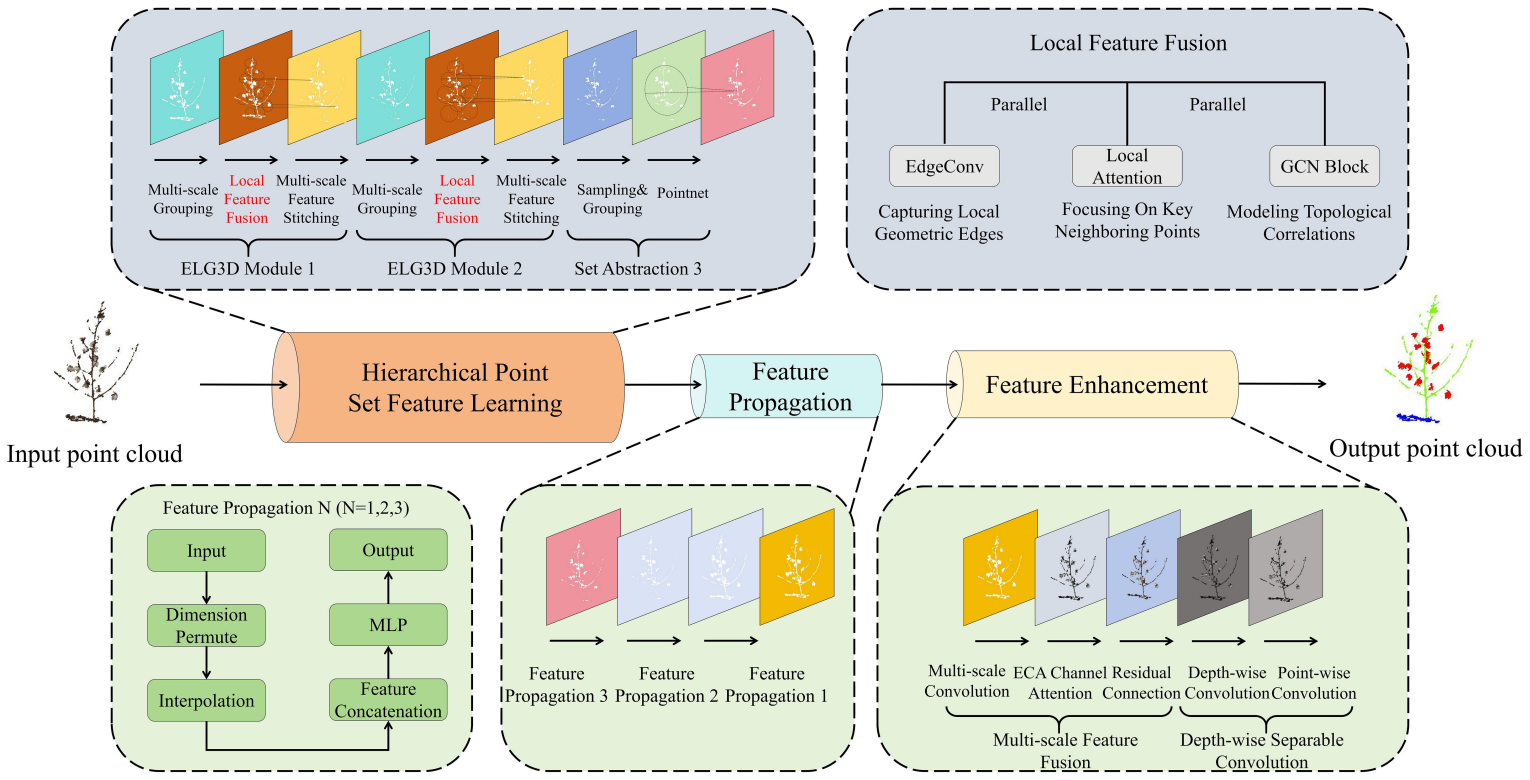
The overall architecture of the ELGCot3D model. This architecture redesigns the traditional SetAbstraction module into an ELG3D module with enhanced local point cloud capture capability, and adds a feature enhancement component, significantly improving feature discriminability for cotton phenotypic segmentation.

To address the technical bottlenecks in 3D phenotypic segmentation of cotton, this paper proposes ELGCot3D, a lightweight and high-fidelity segmentation architecture. Focusing on resource-constrained scenarios, this architecture aims to maximize simultaneous improvements in accuracy and computational efficiency. Specifically, it greatly enhances the ability to capture local features of overlapping cotton organs by reconstructing the traditional Set Abstraction module into the ELG3D module. Second, a cotton-specific feature enhancement module is specially designed to streamline model parameters while strengthening feature discriminability, ultimately achieving an efficient balance between performance and computational efficiency. The overall architecture of ELGCot3D is illustrated in **Figure 1**.

### 2.2 ELG3D module

The Set Abstraction (SA) module of the original PointNet++ (Qi et al., 2017b) is the core of low-level feature extraction, with a logic of sampling-grouping-MLP aggregation. It performs multi-scale grouping after dimensionality reduction via Farthest Point Sampling (FPS) and aggregates local features relying on Multi-Layer Perceptron (MLP). However, considering the characteristics of cotton point clouds, such as severe organ occlusion, dense foliage, and the morphological similarity between bolls and leaves, the SA module design has limitations: relying solely on MLP mapping fails to distinguish the edges of occluded regions and is difficult to focus on key features, while the lack of modeling for organ topological correlations results in inadequate feature representation of complex local structures, ultimately compromising segmentation accuracy.

**Figure 2:**
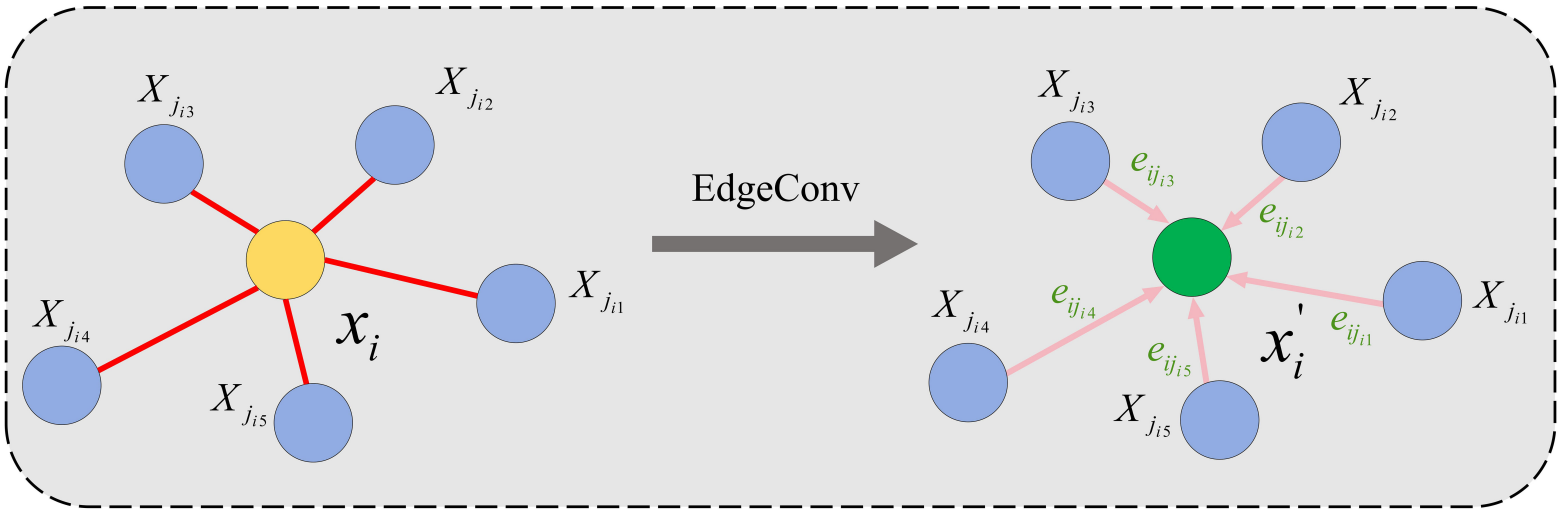
Visualization of the EdgeConv operation. The output of EdgeConv is calculated by aggregating the edge features of all edges associated with each connected vertex.

**Figure 3:**
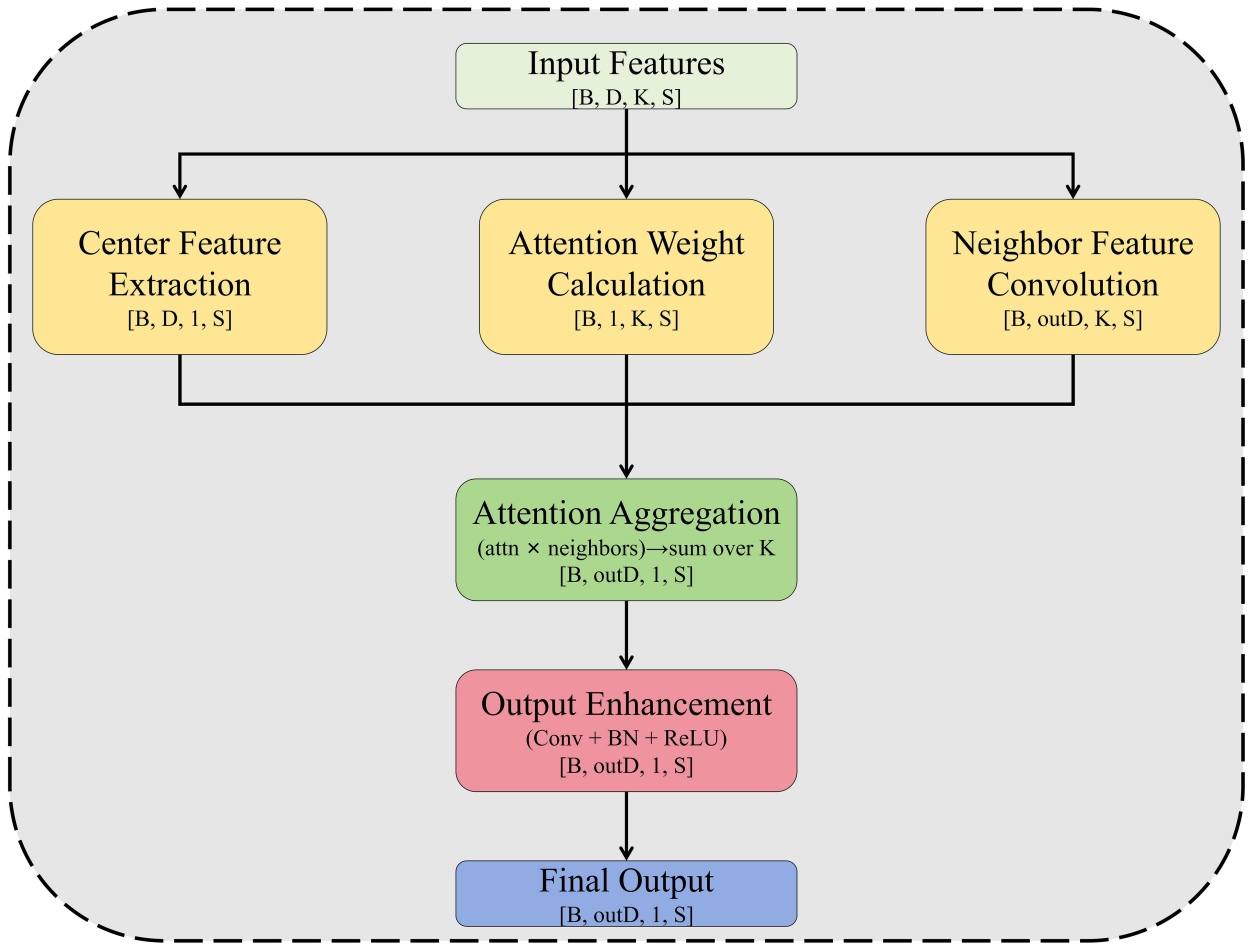
Schematic diagram of the graph convolutional network (GCN) operator’s processing flow and topological correlation modeling. This figure illustrates the core workflow of the GCN operator in the ELG3D module, emphasizing its advantage in modeling the topological correlations of cotton organs. The direction of arrows in the figure indicates the feature flow direction.

To address the above issues, this paper reconstructs the SA module into the EdgeConv-Local Attention-GCN Hybrid 3D Cotton Phenotype Segmentation Module (ELG3D), retaining the advantage of multi-scale grouping while introducing three core operators to enhance feature learning. First, the EdgeConv (Wang et al., 2019) operator is introduced (**Figure 2**) to accurately capture local geometric structures of cotton organs, such as leaf serrations and spherical cotton boll contours. Second, a Local Attention (Harley et al., 2017) operator is designed to enhance the model’s focus on key segmentation-related features (e.g., boll-petiole connections, leaf edge inflection points) and suppress redundant information in background or overlapping regions, improving feature discriminability under complex morphologies. Third, a Graph Convolution (GCN) (Kipf, 2016) operator is embedded (**Figure 3**) to strengthen topological correlation modeling, enabling accurate capture of inter-organ connections (e.g., leaf-main stem attachment, boll-fruiting branch connection) and enriching semantic dependence of local features, thus enhancing segmentation performance in complex scenarios like interlaced branches.

The three operators process multi-scale grouped neighborhood features in parallel, with EdgeConv capturing geometric details, Local Attention emphasizing key region weights, and GCN modeling organ topological correlations to generate three differentiated feature types. First, residual fusion is applied to features of the same scale to retain core information without attenuation, followed by integration of cross-scale fused features. Finally, 1×1 convolution unifies dimensions and strengthens cross-scale correlations, forming the complete ELG3D module. Compared with the original SA module’s single feature output, the module’s enhanced local features integrate geometric, weight, and topological information with multi-scale expression capability, significantly boosting feature discriminability and laying a core foundation for subsequent high-level feature enhancement and high-precision cotton organ segmentation.

### 2.3 Cotton-specific feature enhancement module

Cotton point cloud semantic segmentation faces three core challenges that drive the design of the Cotton-Specific Feature Enhancement Module. First, there is significant organ scale heterogeneity, organs such as cotton bolls, leaves, and main stems vary greatly in size and morphology, making traditional single-scale feature extraction difficult to simultaneously adapt to the representation needs of different organs. Second, severe feature-background confusion exists. In scenarios with dense branches and leaves, point clouds of target organs are easily submerged by redundant backgrounds, which makes it hard to accurately capture key semantic information such as the connection between cotton bolls and petioles and leaf edge contours. Third, balancing lightweighting and representativeness is challenging. Simply stacking network layers to improve feature expression leads to a sharp increase in parameters, failing to meet the efficiency requirements of crop phenotyping. Meanwhile, lightweight designs tend to lose fine-grained structural information of point clouds.

**Figure 4:**
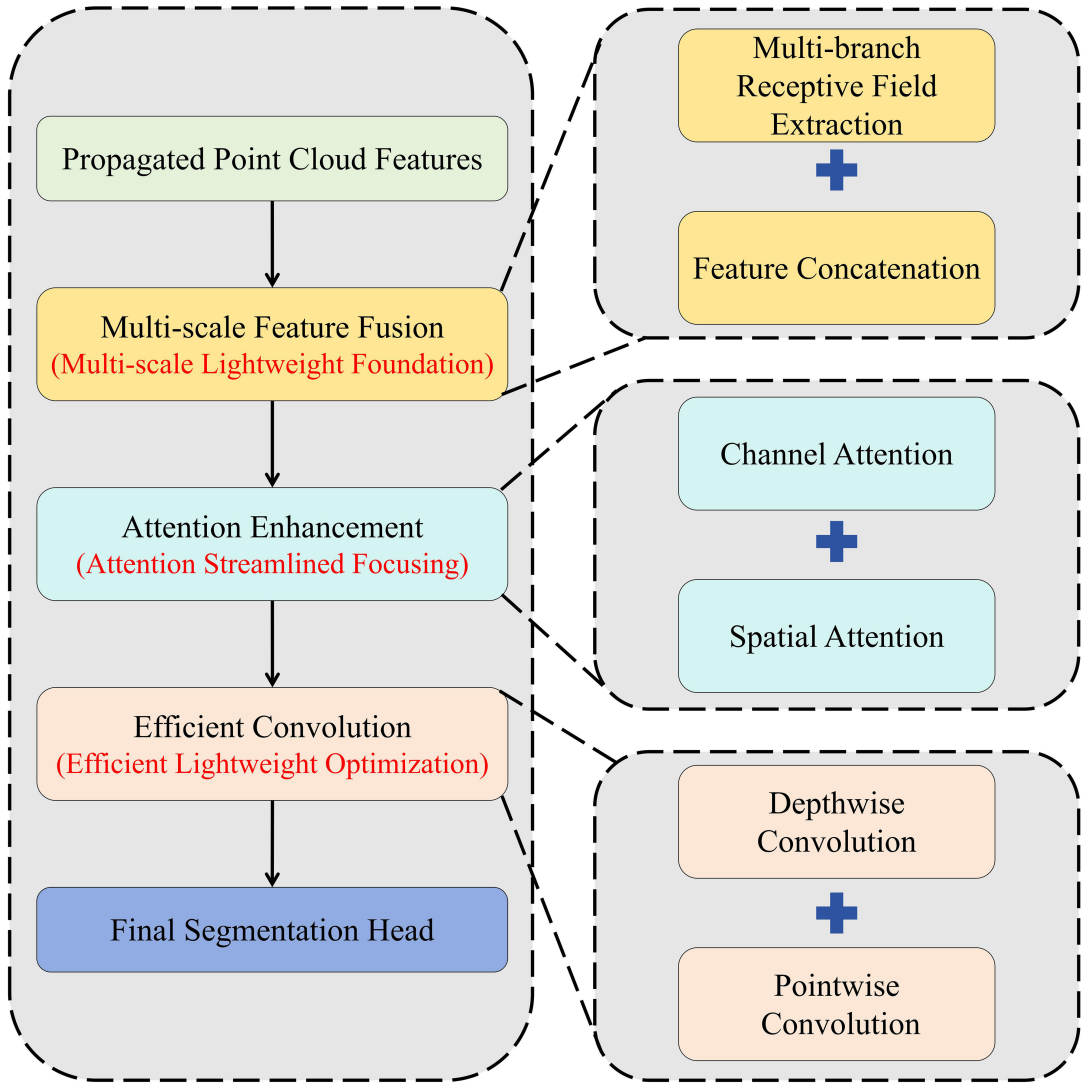
Architecture of the cotton-specific feature enhancement module. The direction of arrows in the figure indicates the feature flow direction, and dashed boxes mark the core innovations of the module. It forms a hierarchical complementarity with the ELG3D module (Section 2.2), ranging from local purification to global enhancement.

The proposed Cotton-Specific Feature Enhancement Module is based on multi-scale feature fusion, centered on attention mechanism, and supported by efficient convolution, with its overall architecture illustrated in **Figure 4**. It is particularly important to note that there are significant differences in both action levels and targets between the attention mechanism of this module and the Local Attention in the ELG3D module (Section 2.2). The Local Attention in ELG3D focuses on fine-grained feature selection between points in local neighborhoods, while the dual-channel attention of this module acts at the global feature level. Specifically, the module first performs multi-branch parallel processing on the point cloud features after Feature Propagation. It extracts receptive field features of multiple scales through branches with different convolution kernel sizes to cover the scale differences of cotton organs, and forms a full-scale feature base after feature concatenation and integration. On this basis, the introduced dual-channel attention mechanism conducts global feature selection. Channel attention enhances the discriminability of different semantic categories (such as cotton bolls, stems, and leaves) in the channel dimension by modeling the dependencies between feature channels. Spatial attention focuses on the distribution pattern of target organs in the entire point cloud space, suppressing feature interference from redundant background points. The synergy of the two mechanisms achieves accurate enhancement of key information in both global semantic and spatial dimensions. Finally, to balance computational efficiency and representational capability, the module adopts depthwise separable convolution instead of traditional convolution operations. It first performs independent convolution on each channel through depthwise convolution to retain fine-grained structures, then completes information interaction between channels via pointwise convolution. This reduces parameters and computational complexity by more than 50% while ensuring the discriminability of enhanced features. It forms a hierarchical complementarity with the ELG3D module, from local point-level feature purification to global feature optimization, providing efficient and accurate feature input for subsequent segmentation heads.

### 2.4 Innovative training strategy for cotton 3D point cloud distribution

The ELGCot3D model faces two core bottlenecks in training for cotton 3D point cloud segmentation tasks, limiting its segmentation accuracy and robustness. On one hand, training stability is insufficient. Traditional schemes struggle to adapt to the fine-grained feature learning needs of cotton point clouds. A single optimizer cannot balance convergence speed and gradient stability, making it prone to gradient explosion or vanishing. The learning rate scheduling with fixed step decay (StepLR) causes abrupt learning rate changes, leading to rough parameter adjustments in the late training stage and oscillating segmentation errors. On the other hand, generalization ability in complex scenarios is limited. Practical cotton point cloud collection involves issues such as rotational posture differences, point cloud missing, and occlusion. However, traditional data augmentation is not designed for the characteristics of 3D point clouds, resulting in insufficient data distribution diversity. This makes the model poorly adaptable to complex scenarios and unable to stably extract edge details and local features.

To address the aforementioned issues, a coordinated improvement plan is designed from three aspects: optimizer, learning rate scheduling, and data augmentation. In the optimizer part, this paper adopts a custom MuSGD optimizer, which combines the momentum update of Momentum-SGD with the exponential moving average of squared gradients from RMSProp to construct a hybrid optimization strategy with adaptive scaling capability, with the corresponding formulas shown in Equations 1–5. Ideologically, this method shares conceptual similarities with Muon’s “update-norm consistency” but removes its expensive orthonormalization constraint, thus maintaining a lightweight computational overhead, and relying on a momentum buffer and a squared-gradient accumulator, MuSGD can effectively smooth gradient changes and mitigate gradient explosion or oscillation, thereby enhancing the stability of the training process.

(1) $$g_{t}=\nabla_{p}L_{t}+\lambda p_{t}$$

(2) $$m_{t}=\beta m_{t-1}+g_{t}$$

(3) $$v_{t}=\mu v_{t-1}+g_{t}^{2}$$

(4) $$d_{t}=\sqrt{v_{t}}+\epsilon$$

(5) $$p_{t+1}=p_{t}-\eta \frac{m_{t}}{d_{t}}$$

During the MuSGD optimization process, *η* denotes the learning rate, which is used to adjust the update magnitude of model parameters in each iteration; *β* is the momentum coefficient, which performs exponential moving average on the first-order gradient term to construct a momentum buffer and accelerate the consistency of the descent direction; *µ* is the exponential decay factor of the squared gradient accumulation term, which determines the smoothness of adaptive scaling (RMSProp-like scaling); *λ* is the weight decay coefficient, i.e., the parameter of the *L*_2_ regularization term, which is used to constrain the parameter norm and reduce the risk of overfitting; $\epsilon =10^{-8}$ is the numerical stability term, which is used in the adaptive denominator $\sqrt{v_{t}}+\varepsilon$ to avoid division by zero and improve the stability of the training process.

For learning rate scheduling, a cosine annealing strategy is employed to allow the learning rate to smoothly decrease along a cosine curve—ensuring fast parameter updates in the early stage and achieving fine-tuning in the later stage to reduce error oscillation. In terms of data augmentation, three types of targeted augmentation operations are designed: First, introducing random Z-axis rotation to enhance the model’s adaptability to different postures of cotton 3D point clouds by randomly adjusting the rotation angle of point clouds in the plane. Second, implementing a random point dropout strategy to randomly eliminate part of the point cloud data with a maximum proportion of 10%, simulating point cloud missing scenarios that may occur in actual collection processes and improving the model’s anti-interference generalization ability. Third, adopting a mirror flipping operation to perform mirror transformation along the X-axis or Y-axis with a 50% probability, further enriching the spatial distribution characteristics of training data; the three work synergistically to improve the model’s convergence efficiency, adaptability to complex scenarios, and segmentation accuracy.

## 3 Materials

### 3.1 Point cloud dataset

To comprehensively and multi-dimensionally verify the performance and adaptability of the proposed model, this paper adopts a dual verification strategy combining general public datasets and cotton-specific 3D point cloud datasets, aiming to cover the evaluation requirements of cross-crop generalization and adaptability to cotton-specific scenarios.

**Figure 5:**
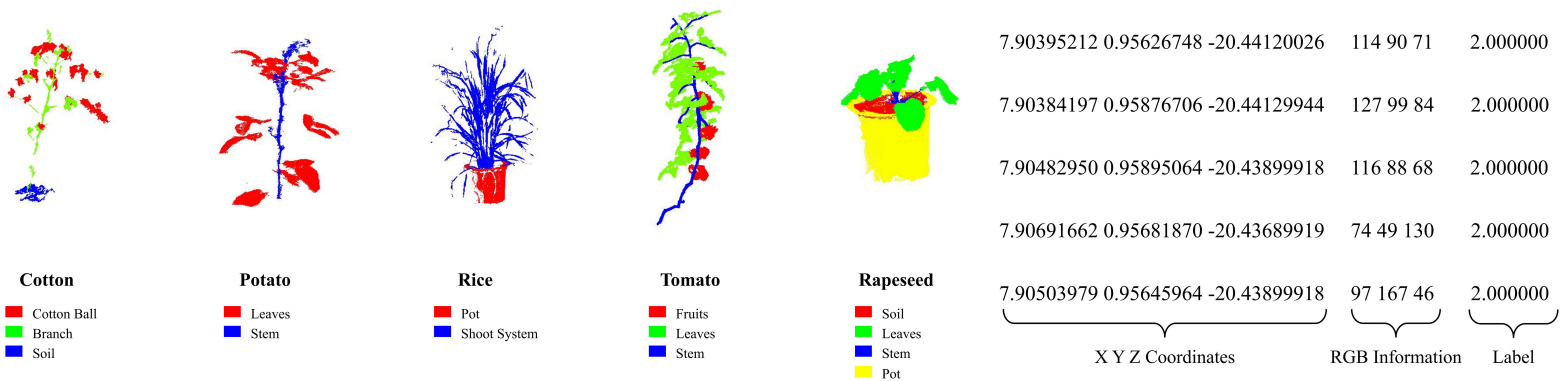
Examples of the public Crops3D multi-crop mixed dataset.

First, systematic experiments are conducted on the public multi-crop mixed Crops3D dataset (Zhu et al., 2024), which contains abundant multi-crop point cloud samples. Partial sample examples are shown in **Figure 5**. Among them, cotton, potato, rice, and rapeseed point clouds are collected via Terrestrial Laser Scanning (TLS), with 176 individual plants, 118 individual plants, 84 individual plants, and 150 individual plants respectively. Tomato point clouds are collected through Structure from Motion (SfM) technology, totaling 83 individual plants.

**Figure 6:**
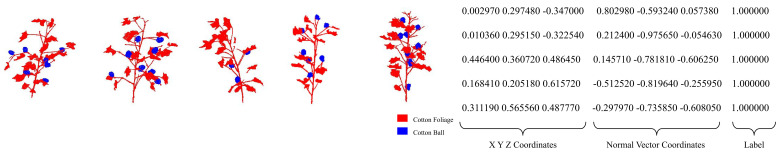
Examples of the cotton data cotton-specific point cloud dataset.

Second, ablation experiments are conducted on the cotton subset of the Crops3D dataset, while generalization experiments use the Cotton data cotton-specific point cloud dataset (https://github.com/UGA-BSAIL/Cotton_plants_with_foliage). Cotton data contains 80 individual plant point clouds, divided into two categories: Cotton Ball and Cotton Foliage. It focuses on covering typical challenging scenarios in cotton segmentation such as dense foliage occlusion and organ scale heterogeneity, which can specifically verify the model’s segmentation accuracy and environmental robustness. The dataset addresses the limitation of general datasets in covering cotton-specific phenotypes and scenarios, with partial sample examples shown in **Figure 6**.

### 3.2 Experimental details

To ensure the fairness of experimental comparisons, credibility of results, and reproducibility, all comparative models are trained from scratch without pre-trained weights, with experiments conducted on a server equipped with an NVIDIA GeForce RTX 4090 GPU (Santa Clara, CA, USA) and implemented based on the PyTorch v2.2.2 deep learning framework. At the input data level, considering that the Crops3D dataset contains both XYZ coordinates and RGB color parameters, while the specialized cotton point cloud dataset Cotton data includes both XYZ coordinates and Normal vector coordinates, all models are uniformly trained with only XYZ coordinates as input features to eliminate interference caused by input data differences. Regarding training configurations, key hyperparameters are summarized in **Table 1**, and unless otherwise specified, all experimental datasets are fixedly split into training, validation, and test sets at a 7:2:1 ratio to ensure different models are evaluated under consistent data distribution conditions.

**Table 1:** Key hyperparameters used for training all models.

Hyperparameter	Value
Optimizer	MuSGD
Base Learning Rate	1 × 10^−3^
Npoint	2048
Batch Size	8
Number of Epochs	300

### 3.3 Evaluation metrics

This paper employs two types of evaluation metrics, namely segmentation accuracy and model efficiency, to assess the model. For comprehensive evaluation of segmentation accuracy, mean Intersection over Union (mIoU) and Overall Accuracy (OA) are adopted as specific metrics, with their calculation formulas shown in Equations 6, 7.

(6) $$\text{mIoU}=\frac{1}{M}\sum_{i=1}^{M}\frac{{\text{TP}}_{i}}{{\text{TP}}_{i}+{\text{FP}}_{i}+{\text{FN}}_{i}}$$

(7) $$\text{OA}=\frac{\text{TP}+\text{TN}}{\text{TP}+\text{TN}+\text{FP}+\text{FN}}$$

In the formula, M in the formulas denotes the total number of categories in the segmentation task. True Positive (TP) refers to the number of samples that actually belong to the positive class and are correctly classified as the positive class. True Negative (TN) is the number of samples that actually belong to the negative class and are correctly classified as the negative class. False Positive (FP) represents the number of samples that actually belong to the negative class but are incorrectly classified as the positive class. False Negative (FN) denotes the number of samples that actually belong to the positive class but are incorrectly classified as the negative class.

Meanwhile, to measure the model efficiency, we report the total number of trainable parameters in the model (Params) and Giga Floating-Point Operations Per Second (GFLOPs). Params refers to the total number of trainable parameters in the model, measured in millions (M), and reflects the model’s size and memory footprint. GFLOPs measures the computational complexity of a single forward pass and serves as a key indicator of inference speed.

## 4 Results

### 4.1 Comparison with state-of-the-art methods

On the Crops3D validation set, we conducted comparative evaluations of ELGCot3D against various classic and cutting-edge target detectors, with results shown in **Table 2**. The model achieved 76.7% mIoU and 85.8% OA, increasing by 5.1% and 4.2% respectively compared with the PointNet++ baseline model. It has only 0.86 M parameters, a reduction of 50.1% compared with PointNet++, and a computational complexity of 2.41 GFLOPs, a reduction of 50.7% compared with PointNet++. All performance metrics outperform high-performance models such as PointMLP. This result is attributed to the rationality of the task-aware design approach in this paper. Instead of relying on computational power stacking, the model optimizes the local feature extraction module to specifically adapt to core segmentation challenges in cotton such as dense foliage occlusion and organ scale heterogeneity. While streamlining redundant parameters, it effectively retains key cotton phenotypic information, ultimately achieving the coordinated improvement of accuracy and efficiency under a lightweight architecture. This provides a practical and deployment-feasible solution for cotton segmentation tasks.

**Table 2:** Performance comparison with state-of-the-art models on crops3D validation set.

Method	Species	mIoU (%)	OA (%)	Params (M)	GFLOPs
PointNet	Cotton	57.1	70.4	8.33	5.76
	Potato	73.7	95.2		
	Rice	93.5	96.8		
	Tomato	69.3	89.6		
	Rapeseed	65.6	90.9		
PointCNN	Cotton	74.9	83.6	2.50	5.23
	Potato	83.6	96.3		
	Rice	94.9	97.1		
	Tomato	75.2	90.7		
	Rapeseed	75.6	90.2		
CurveNet	Cotton	71.3	81.1	2.50	15.77
	Potato	80.2	94.9		
	Rice	95.1	97.8		
	Tomato	77.3	89.9		
	Rapeseed	75.4	89.2		
DGCNN	Cotton	69.6	79.3	2.80	6.35
	Potato	86.5	97.1		
	Rice	96.7	98.8		
	Tomato	53.8	72.4		
	Rapeseed	77.1	91.3		
PointMLP	Cotton	71.6	80.2	13.20	26.38
	Potato	77.3	93.2		
	Rice	95.1	97.7		
	Tomato	65.8	83.6		
	Rapeseed	72.1	85.1		
PointNet++ (Baseline)	Cotton	71.6	81.6	1.70	4.89
	Potato	84.9	97.1		
	Rice	97.1	98.6		
	Tomato	78.5	91.2		
	Rapeseed	75.6	94.8		
ELGCot3D (Ours)	Cotton	76.7	85.8	0.86	2.41
	Potato	87.9	97.9		
	Rice	98.1	99.1		
	Tomato	80.6	92.4		
	Rapeseed	80.1	95.4		

### 4.2 Ablation study

To verify the effectiveness of each proposed core component, this paper conducts systematic ablation experiments on the cotton subset of the Crops3D dataset. Using PointNet++ as the baseline model, the performance of the model under different component combinations is compared among the proposed ELG3D module, specialized feature enhancement module, and innovative training strategy to quantify the value of each component. As shown in **Table 3**, the experimental results confirm that each proposed component not only can independently bring significant gains to the model’s segmentation accuracy but also can further improve the overall performance through synergistic adaptation between components, fully verifying the necessity and synergy of each component’s design.

**Table 3:** Ablation performance on Crops3D validation set.

Configuration	mIoU (%)	ΔmIoU	OA (%)	Params (M)
(A) Baseline (PointNet++)	71.6	–	81.6	1.73
(B) Baseline + ELG3D Module	75.3	+3.7	84.4	2.13
(C) Baseline + Feature Enhancement Module	74.1	+2.5	83.1	0.77
(D) Baseline + Innovative Training Strategy	72.7	+1.1	82.9	1.73
(E) ELGCot3D (B+C+D)	76.7	+5.1	85.8	0.86

Quantifying contributions of core modules and training strategy.

Among them, the ELG3D module achieves the optimal performance improvement, with mIoU increased by 3.7% and OA by 2.8%, verifying our hypothesis that the module can more efficiently capture fine-grained features and spatial correlation information of cotton organs by reconstructing the traditional Set Abstraction module and optimizing local feature fusion logic, laying a core foundation for improved segmentation accuracy. Meanwhile, integrating only the specialized feature enhancement module increases mIoU by 2.5% while further reducing the number of parameters compared with the baseline model, highlighting its strong adaptability to the semantic characteristics of cotton 3D point clouds. It strengthens organ-level information of cotton bolls and foliage through multi-scale feature fusion and precisely focuses on core semantic regions with a dual-channel attention mechanism, effectively suppressing background redundant interference and reducing unnecessary computational redundancy to balance accuracy improvement and lightweight requirements. Additionally, adopting the innovative training strategy increases mIoU by 1.1%, underscoring its precise adaptability to the data characteristics of cotton 3D point clouds; compared with general training schemes, it optimizes the model’s learning focus on complex scenarios by matching the point cloud distribution law of cotton organs, effectively reducing segmentation deviations caused by mismatched data characteristics and providing reliable training-level support for the stable improvement of model performance.

The aforementioned three core components are synergistically integrated to finally construct the complete ELGCot3D 3D cotton point cloud segmentation model. Experimental results show that compared with the baseline model, the total mIoU gain of this model reaches 5.1%, while the number of parameters is reduced by 50.1% compared with the baseline model, ultimately only 0.86M. The above results fully verify that the contributions of each core component have significant complementarity, jointly establishing an efficient and lightweight 3D point cloud segmentation framework that balances segmentation accuracy and computational efficiency, which can effectively adapt to the practical application needs of cotton point cloud segmentation.

### 4.3 Qualitative analysis

**Figure 7:**
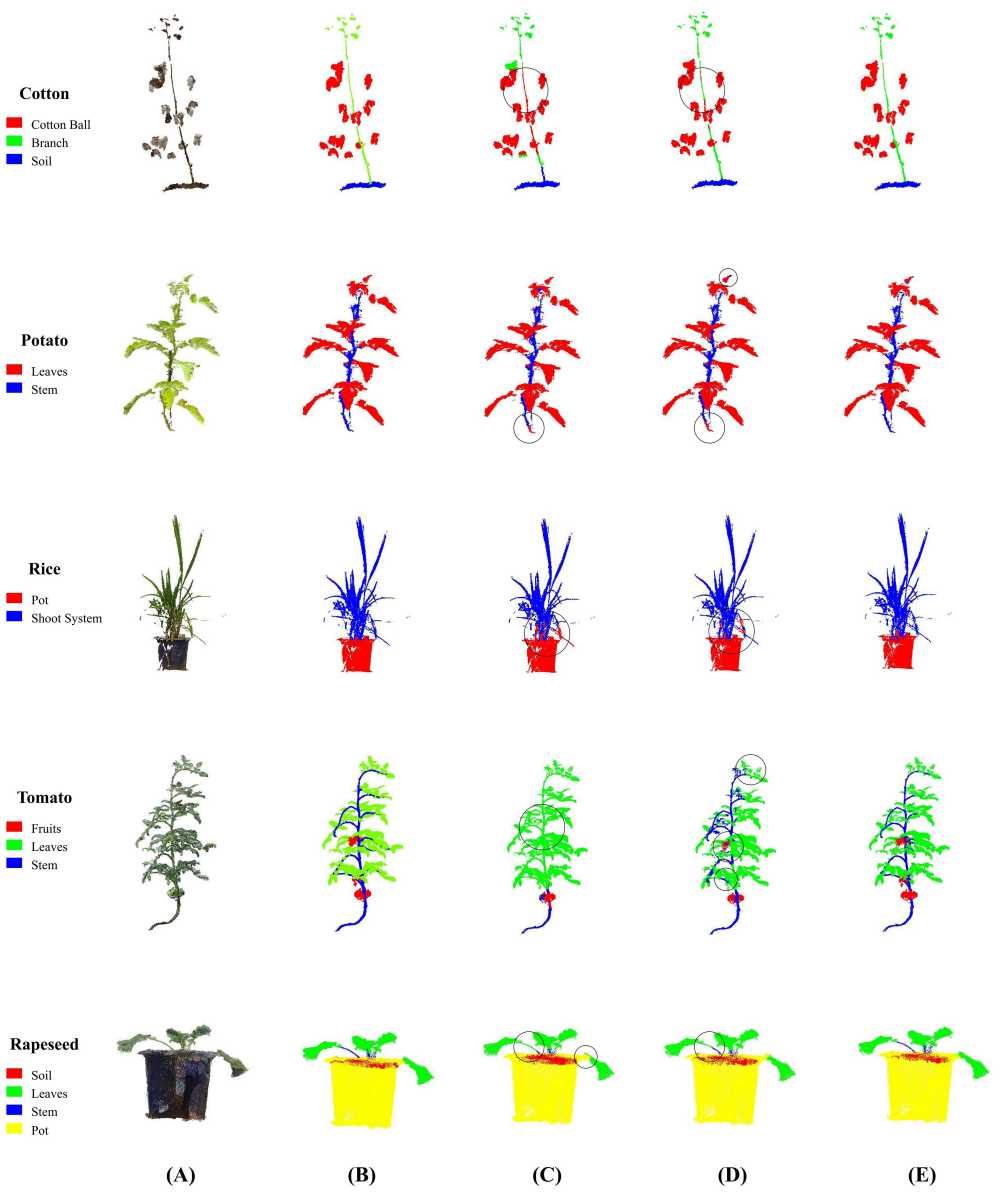
Visual comparison results of multi-crop segmentation performance on the Crops3D dataset. Compared with the PointNet model and PointNet++ baseline model, ELGCot3D achieves more accurate segmentation results and more complete detail restoration—especially at fine structures such as crop foliage junctions, with clearer segmentation boundaries and lower misclassification rates. **(A)** Point Cloud. **(B)** GT. **(C)** PointNet. **(D)** PointNet++ (baseline). **(E)** ELGCot3D.

To intuitively understand the advantages of ELGCot3D, we conducted a qualitative comparison. Regarding the segmentation results, **Figure 7** shows the segmentation outputs of ELGCot3D on point cloud samples of five crops (Cotton, Potato, Rice, Tomato, Rapeseed) covered by the Crops3D dataset, and the model exhibits outstanding performance across different crop scenarios. Specifically, it achieves more accurate segmentation of error-prone fine structures at the junctions between cotton foliage and bolls, and can clearly define organ boundaries to avoid structural adhesion issues. Meanwhile, it recognizes key phenotypic organs of crops more completely, showing significant improvement over the small target missing problem of the PointNet++ baseline model. In addition, facing occlusion scenarios such as dense overlapping of tomato leaves and intertwined canopy branches and leaves of rapeseed, the model has higher robustness—it can effectively distinguish the contours of occluded organs to reduce false negative annotations. Moreover, in areas such as junctions between rice stems and soil, and areas where rapeseed plants are mixed with the environmental background, it can completely suppress background redundant point clouds and reduce false positive annotations, resulting in higher consistency between segmentation results and the ground truth (GT). These visual results correspond with quantitative improvements, intuitively confirming that ELGCot3D can significantly reduce false negative and false positive annotations, and fully verifying its good adaptability to multi-crop point cloud segmentation scenarios.

### 4.4 Generalization analysis

For comprehensive validation of ELGCot3D’s generalization capabilities and preventing its performance from being confined to a single dataset, this paper further conducts experiments on the Cotton data specialized cotton point cloud dataset. It specifically evaluates the model’s generalization performance in cotton-specific scenarios to verify its cross-dataset adaptability and task adaptability.

On the Cotton_data dataset covering different growth stages and morphological differences, ELGCot3D still exhibits segmentation performance exceeding all comparative baseline models. As shown in **Table 4**, its mIoU and OA reach 80.4% and 94.2%, respectively, significantly outperforming baseline models such as PointNet++. This result fully proves that the model has excellent environmental adaptability, it can stably adapt to the appearance, morphology, and complex background scenarios of different cotton plants, effectively reducing false positive and false negative errors during segmentation. Meanwhile, both the number of parameters and computational complexity of ELGCot3D are controlled within 50% of those of the baseline model, achieving ultra-lightweight while ensuring high-precision segmentation. This dual advantage of high precision and low overhead provides key support for the reliable deployment of the model in practical scenarios such as field portable devices and real-time phenotypic monitoring, fully meeting the actual application needs of cotton point cloud segmentation tasks.

**Table 4:** Performance of cotton data specialized cotton point cloud dataset.

Method	mIoU (%)	OA (%)
PointNet	42.4	84.7
PointNet++	56.5	86.0
ELGCot3D (Ours)	80.4	94.2

**Figure 8:**
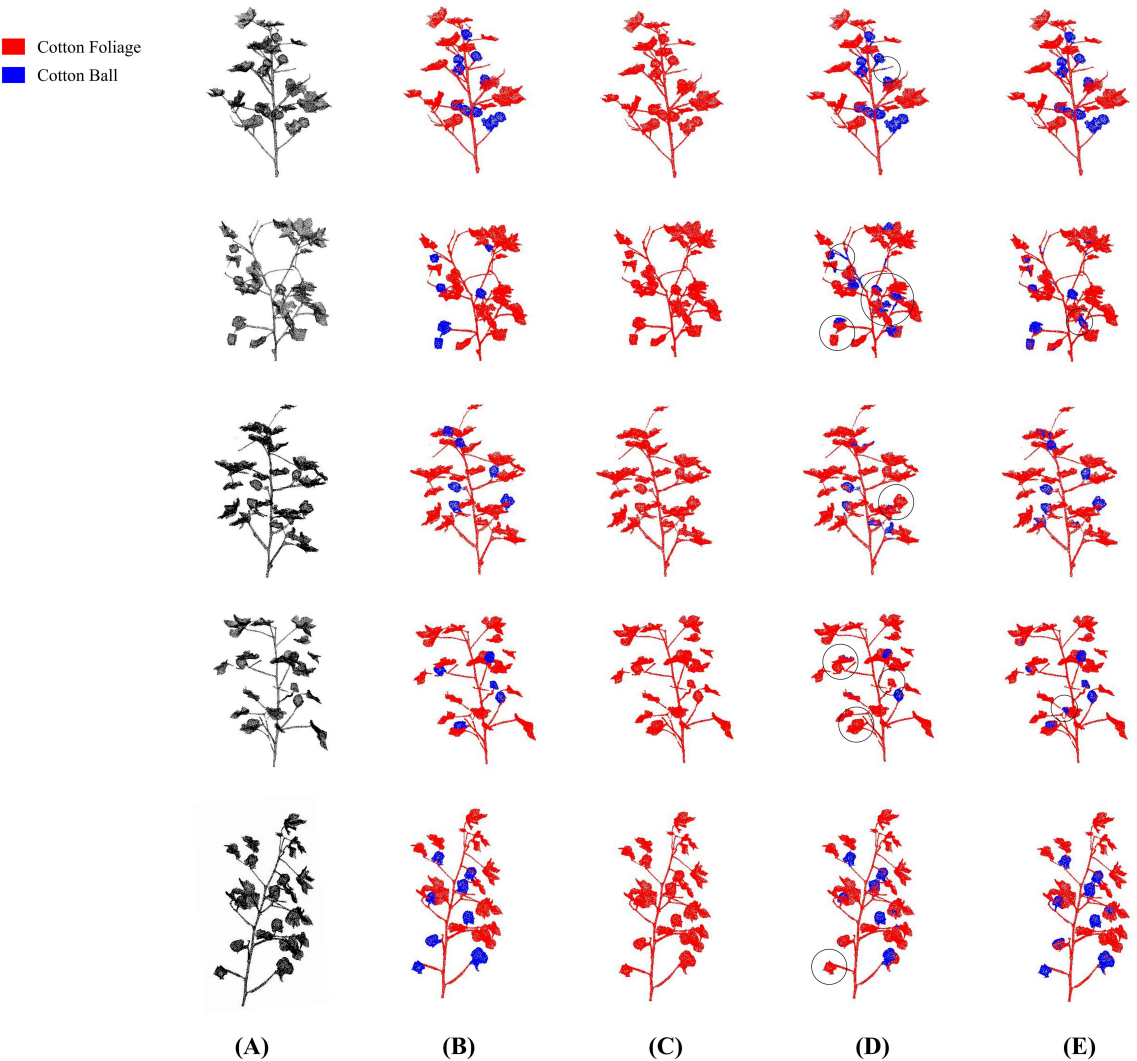
Visual comparison results of segmentation performance on the cotton data specialized cotton point cloud dataset. Compared with the PointNet model and PointNet++ baseline model, ELGCot3D has minor misjudgments only in a few complex areas with dense foliage occlusion or local point cloud missing, but the overall accuracy and detail restoration of its segmentation results are significantly superior. **(A)** Point Cloud. **(B)** GT. **(C)** PointNet. **(D)** PointNet++ (baseline). **(E)** ELGCot3D.

The high precision and low consumption advantages demonstrated by the above quantitative results can be more intuitively verified in the visual segmentation results. **Figure 8** shows the visual segmentation comparison between ELGCot3D and the PointNet, PointNet++ baseline models on the Cotton data specialized cotton point cloud dataset. By intuitively presenting the segmentation effects of different models on the junction areas of cotton bolls and foliage, the detail processing capability of ELGCot3D in complex scenarios can be clearly observed, further confirming its performance superiority in cotton-specific segmentation tasks.

**Figure 9:**
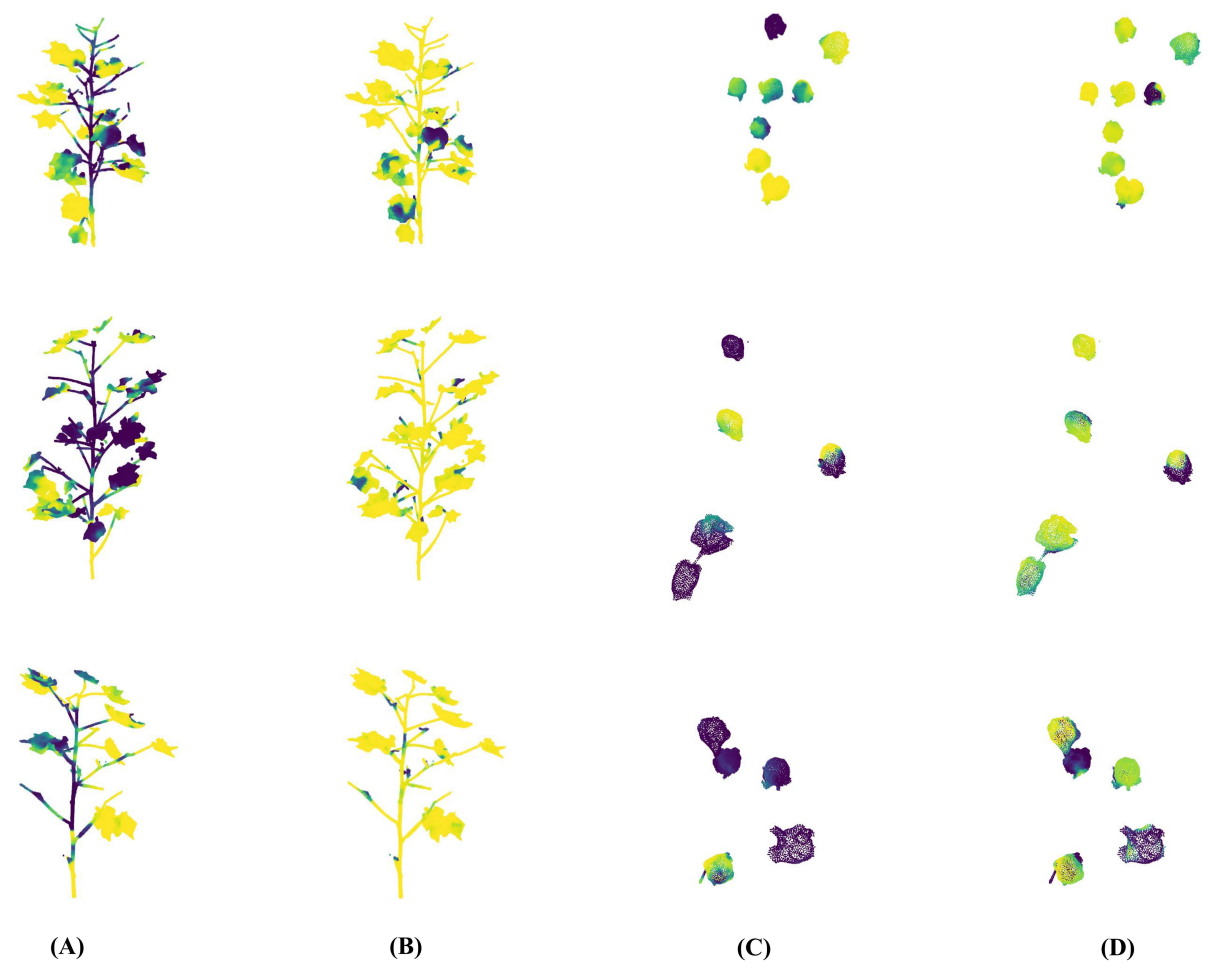
Attention heatmap comparison of “Cotton Foliage - Cotton Ball” on the cotton data specialized cotton point cloud dataset. In the heatmap, a brighter yellow color indicates a higher attention weight of the model on the point cloud, while a darker yellow color indicates a lower weight. **(A)** PointNet++ (Cotton Foliage). **(B)** ELGCot3D (Cotton Foliage). **(C)** PointNet++ (Cotton Ball). **(D)** ELGCot3D (Cotton Ball).

From the perspective of model interpretability, ELGCot3D’s precise segmentation capability for cotton’s core organs fundamentally derives from its attention focus on two key semantic categories: branches and leaves, and bolls. **Figure 9** presents the attention heatmap comparison between ELGCot3D and the PointNet++ baseline model for these core organs, which intuitively visualizes the distribution of model attention weights on each point cloud to reveal semantic capture differences between the models. As shown in the results, ELGCot3D’s attention weights are significantly denser in the effective structural regions of foliage and the surface of cotton bolls, with a higher matching degree to ground truth labels. In contrast, the PointNet++ baseline model exhibits relatively scattered attention distribution and insufficient weight intensity in target organ regions. This difference clearly confirms the effectiveness of the dual-channel attention mechanism in the specialized feature enhancement module—by precisely allocating channel semantic weights and spatial position weights for foliage and cotton bolls, it guides the model to actively focus on core organs and suppress non-target interference, providing direct mechanism-level support for improved segmentation accuracy from the perspective of decision-making logic visualization.

**Figure 10:**
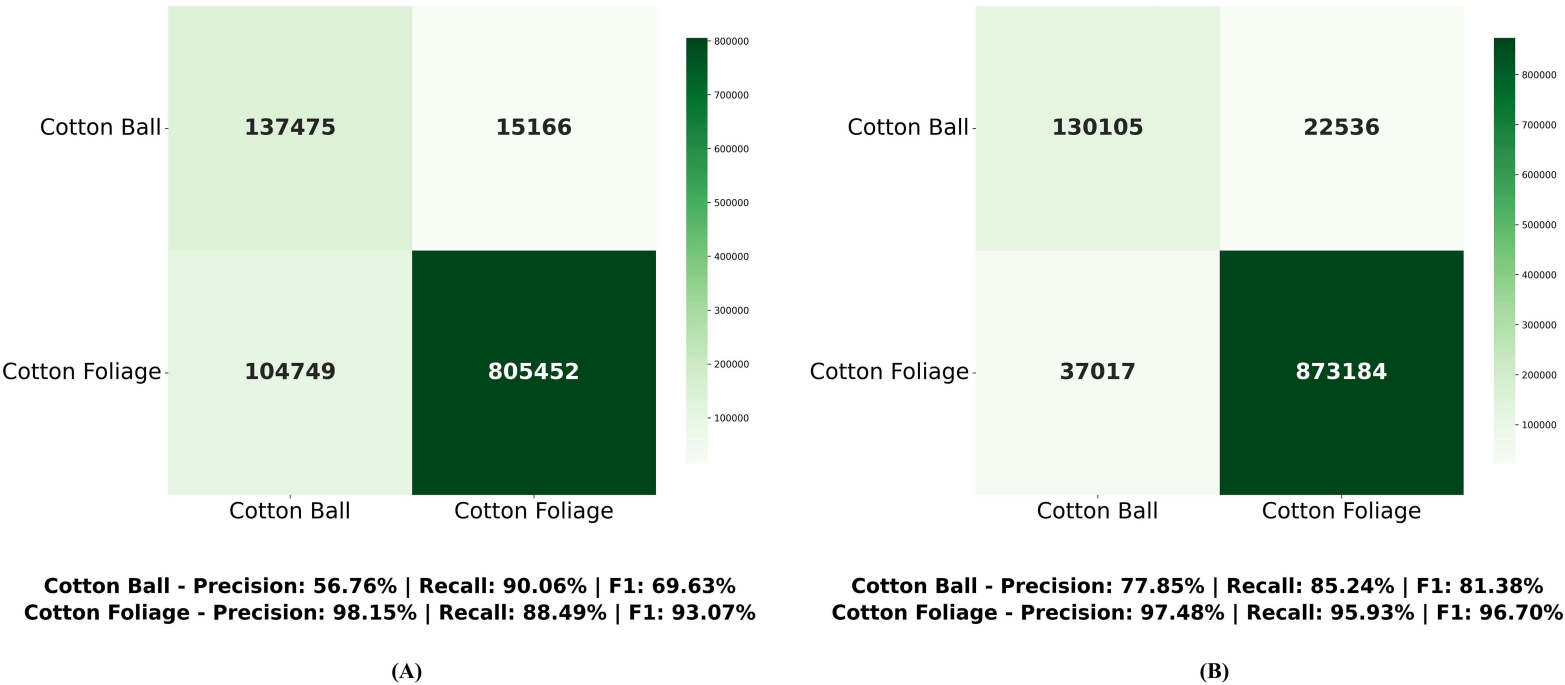
Confusion matrix comparison on cotton data. Comparison of F1-scores shows that ELGCot3D is significantly superior to PointNet++. **(A)** PointNet++ (baseline). **(B)** ELGCot3D.

Finally, **Figure 10** presents the point cloud confusion matrices of ELGCot3D and the baseline model PointNet++ on the Cotton data test set. These matrices intuitively reveal the differences in classification performance between the two methods, clearly showing the correct segmentation and misclassification distribution characteristics of cotton components. Key evaluation metrics including precision, recall, and F1-score are labeled below the matrices. Comprehensive comparison indicates that ELGCot3D exhibits superior segmentation performance.

## 5 Discussion

The ELGCot3D model designed in this paper achieves an effective balance between segmentation accuracy and computational efficiency in 3D cotton point cloud segmentation tasks. It exhibits superior performance over traditional baseline models on both the Crops3D multi-crop dataset and the Cotton data specialized cotton point cloud dataset. Through a multi-operator fusion mechanism, it precisely focuses on effective foliage structures and cotton boll regions, providing technical support for the deployment of field portable devices. However, the model still has limitations: it currently only conducts semantic segmentation for individual cotton plants, and has not yet realized instance segmentation for distinguishing individual organs within the same category. Additionally, the model has not yet been validated with large-scale field point cloud data of densely planted cotton populations, and lacks targeted research on the processing and analysis pipeline for large-area field scenarios, which restricts its direct scalability to practical large-scale cotton phenotyping applications. In the future, the research direction will be further expanded: on the one hand, an instance segmentation branch will be introduced to distinguish individual organs; on the other hand, the model will be extended to the verification and adaptation of large-scale field dense planting scenarios, and a set of efficient point cloud down-sampling and batch processing strategies will be established to promote the practical application of the model in large-area cotton fields. This will provide a more comprehensive technical solution for cotton growth monitoring and yield estimation.

## 6 Conclusion

To address the core pain points of insufficient segmentation accuracy and low deployment efficiency in 3D cotton point cloud segmentation, this paper develops an efficient and lightweight ELGCot3D model, achieving technical breakthroughs through three key improvements. Specifically, it reconstructs the traditional Set Abstraction module into the ELG3D module to enhance the ability to capture fine-grained features of cotton organs. A cotton-specific feature enhancement module integrating multi-scale features and dual-channel attention is designed to precisely focus on core semantic regions, suppress background redundant interference, and reduce computational consumption. Meanwhile, an innovative training strategy adapted to the distribution of cotton point cloud data is proposed to ensure more stable model training. Experimental verification shows that the model performs excellently on both the Crops3D multi-crop dataset and the Cotton data specialized cotton point cloud dataset. Particularly on the Cotton data specialized cotton point cloud dataset, it achieves an mIoU of 80.4% and an OA of 94.2%, with only 0.86M parameters and computational complexity controlled below 50% of the baseline model, realizing a balance between high precision and lightweight. With its lightweight characteristics and efficient inference capability, the model can directly provide technical support for real-time monitoring of cotton phenotypes. Nevertheless, this study has certain limitations that warrant further exploration: the model’s performance is constrained when handling extreme leaf occlusion scenarios where overlapping organs are almost indistinguishable, and its inference efficiency and segmentation stability may decline when processing ultra-large-scale field point cloud data due to the current architecture’s limited scalability for massive data. In the future, relying on the point cloud segmentation model, we will further conduct research on high-throughput phenotypic extraction technology for large-area field cotton plants, optimize the model architecture to address extreme occlusion and large-scale data processing issues, and aim to provide more accurate phenotypic data support for breeders, thereby assisting in optimizing the efficient cotton breeding process and cultivating high-quality new varieties.

## Data Availability

The original contributions presented in the study are included in the article/**Supplementary Material**. Further inquiries can be directed to the corresponding author.
