# Supplementary material for: ELGCot3D: a lightweight 3D cotton point cloud segmentation model based on EdgeConv-Local Attention-GCN and semantic feature enhancement
Source: Front Plant Sci. 2026 Feb 6;17:1765604. doi: 10.3389/fpls.2026.1765604 (PMC12920531; doi:10.3389/fpls.2026.1765604)
Supplement: Supplementary file 1 [file DataSheet1.zip › Figure/7.pdf]

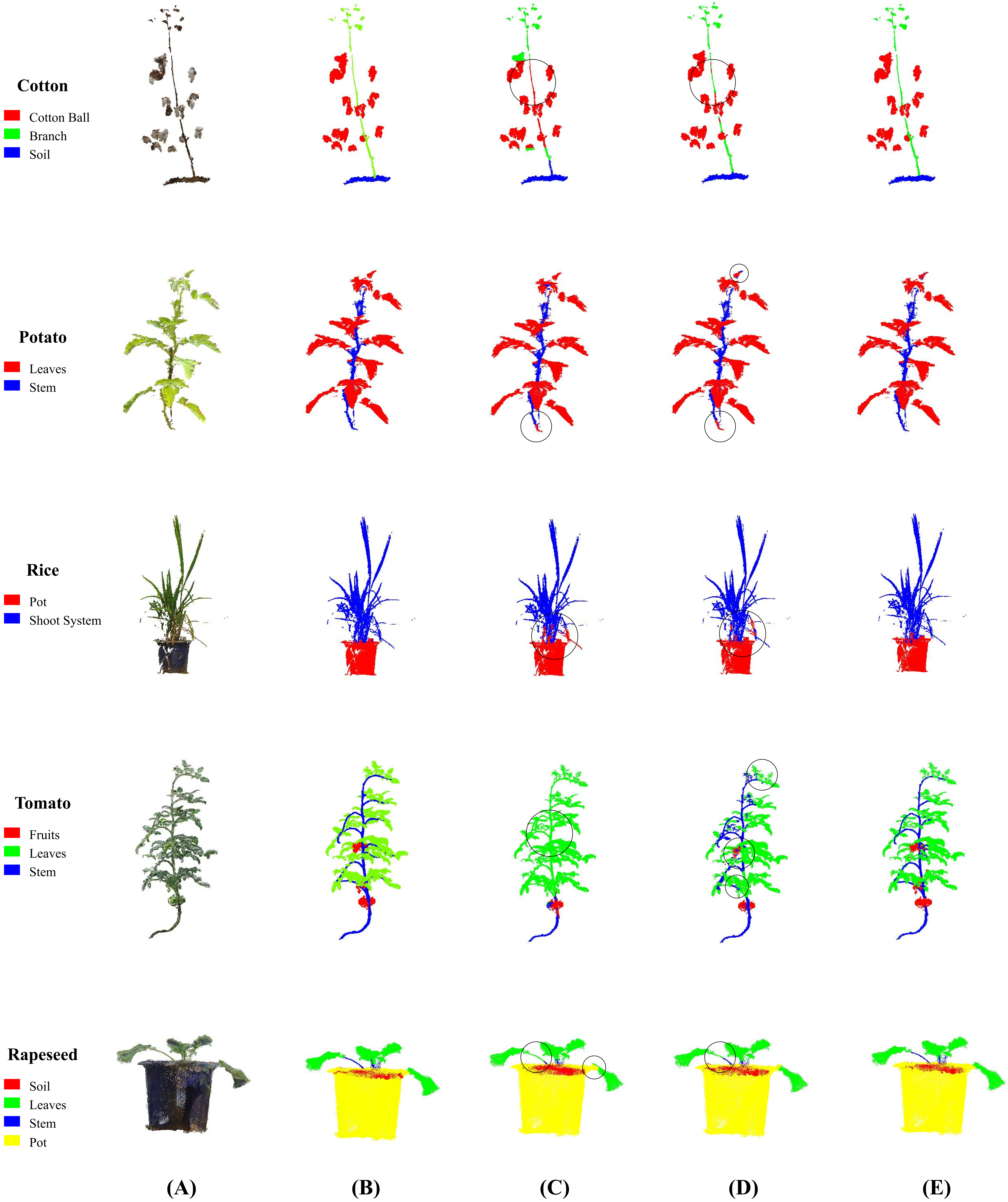

Cotton
Cotton Ball
Branch
Soil
Potato
Leaves
Stem
Rice
Pot
Shoot System
Tomato
Fruits
Leaves
Stem
Rapeseed
Soil
Leaves
Stem
Pot
(A)
(B)
(C)
(D)
(E)
