# Supplementary material for: ELGCot3D: a lightweight 3D cotton point cloud segmentation model based on EdgeConv-Local Attention-GCN and semantic feature enhancement
Source: Front Plant Sci. 2026 Feb 6;17:1765604. doi: 10.3389/fpls.2026.1765604 (PMC12920531; doi:10.3389/fpls.2026.1765604)
Supplement: Supplementary file 1 [file DataSheet1.zip › Figure/10.pdf]

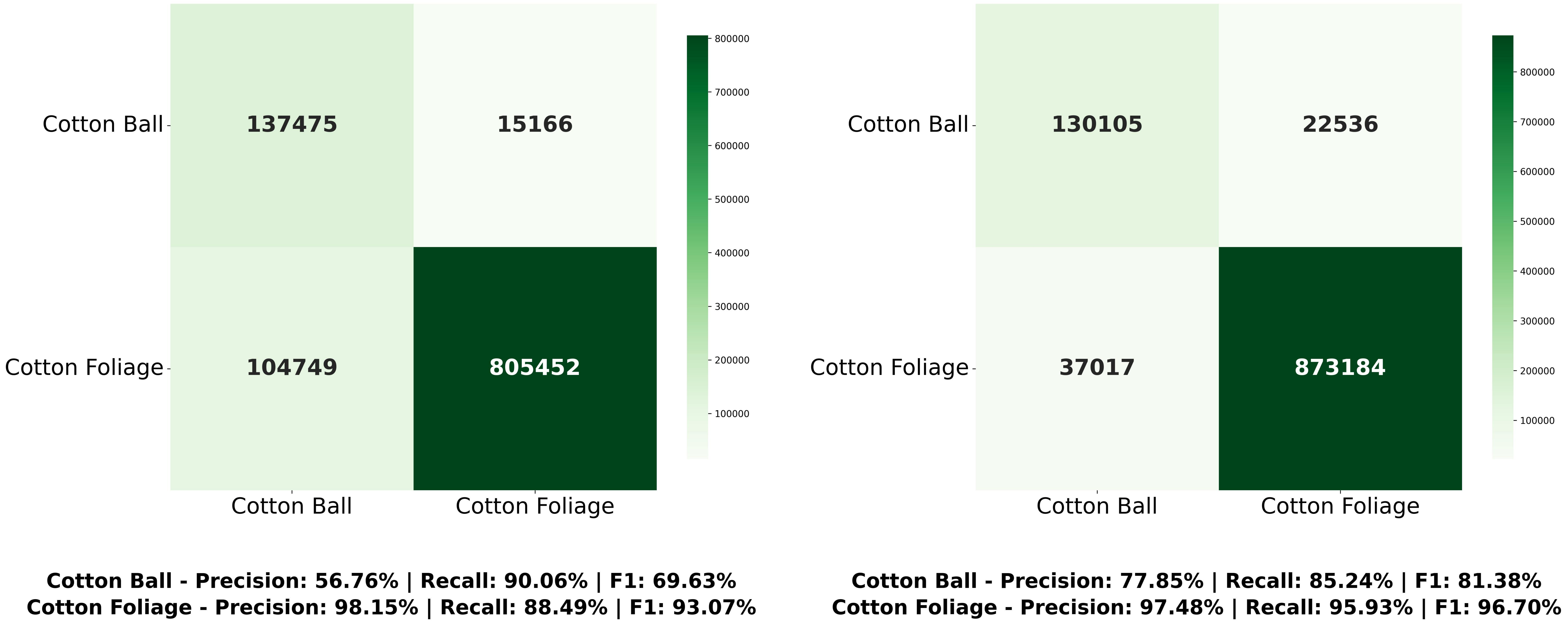
Cotton Ball
Cotton Foliage
137475
15166
104749
805452
Cotton Ball
Cotton Foliage
800000
700000
600000
500000
400000
300000
200000
100000
Cotton Ball - Precision: 56.76% | Recall: 90.06% | F1: 69.63%
Cotton Foliage - Precision: 98.15% | Recall: 88.49% | F1: 93.07%
Cotton Ball
Cotton Foliage
130105
22536
37017
873184
Cotton Ball
Cotton Foliage
800000
700000
600000
500000
400000
300000
200000
100000
Cotton Ball - Precision: 77.85% | Recall: 85.24% | F1: 81.38%
Cotton Foliage - Precision: 97.48% | Recall: 95.93% | F1: 96.70%

(A) (B)
