# Supplementary figures and images for: ELGCot3D: a lightweight 3D cotton point cloud segmentation model based on EdgeConv-Local Attention-GCN and semantic feature enhancement
Source: Front Plant Sci. 2026 Feb 6;17:1765604. doi: 10.3389/fpls.2026.1765604 (PMC12920531; doi:10.3389/fpls.2026.1765604)

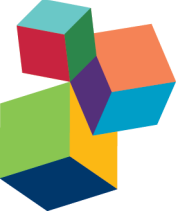

Supplement: Supplementary file 1 [file DataSheet1.zip › logo2.pdf]

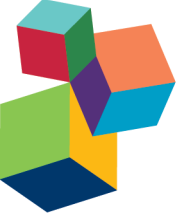

frontiers

Supplement: Supplementary file 1 [file DataSheet1.zip › logo1.pdf]

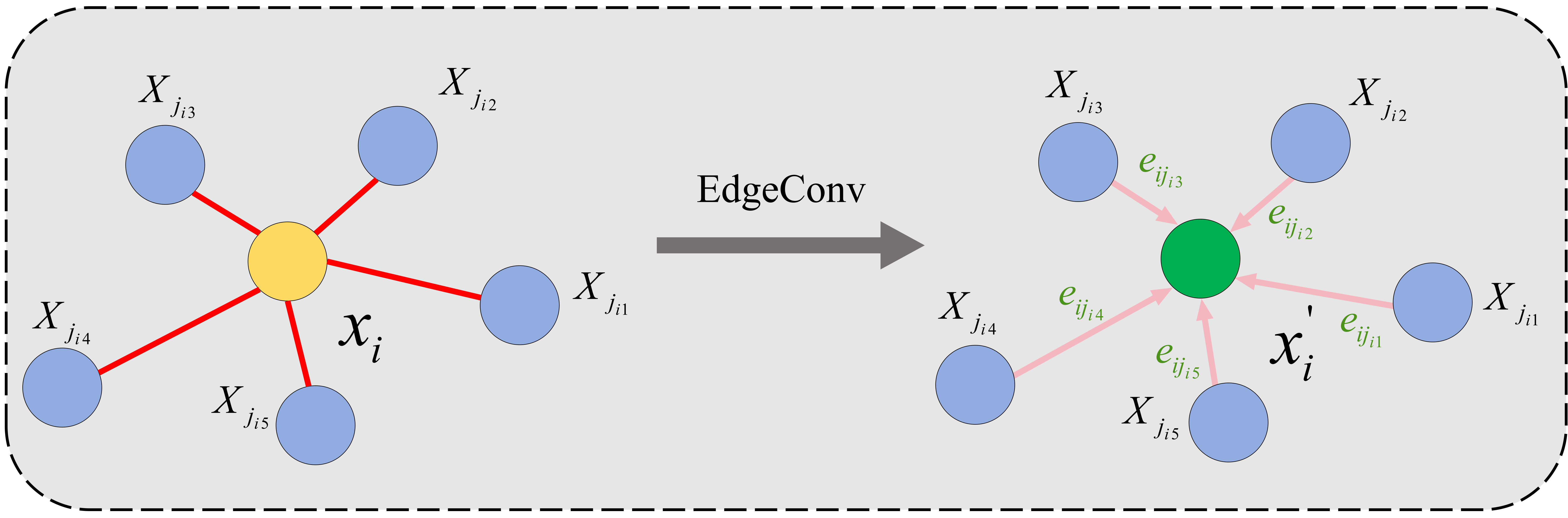

Supplement: Supplementary file 1 [file DataSheet1.zip › Figure/2.pdf]

A

frontiers  
FOR YOUNG MINDS

B

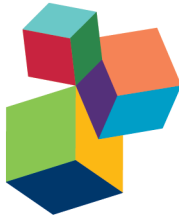

Supplement: Supplementary file 1 [file DataSheet1.zip › logos.pdf]

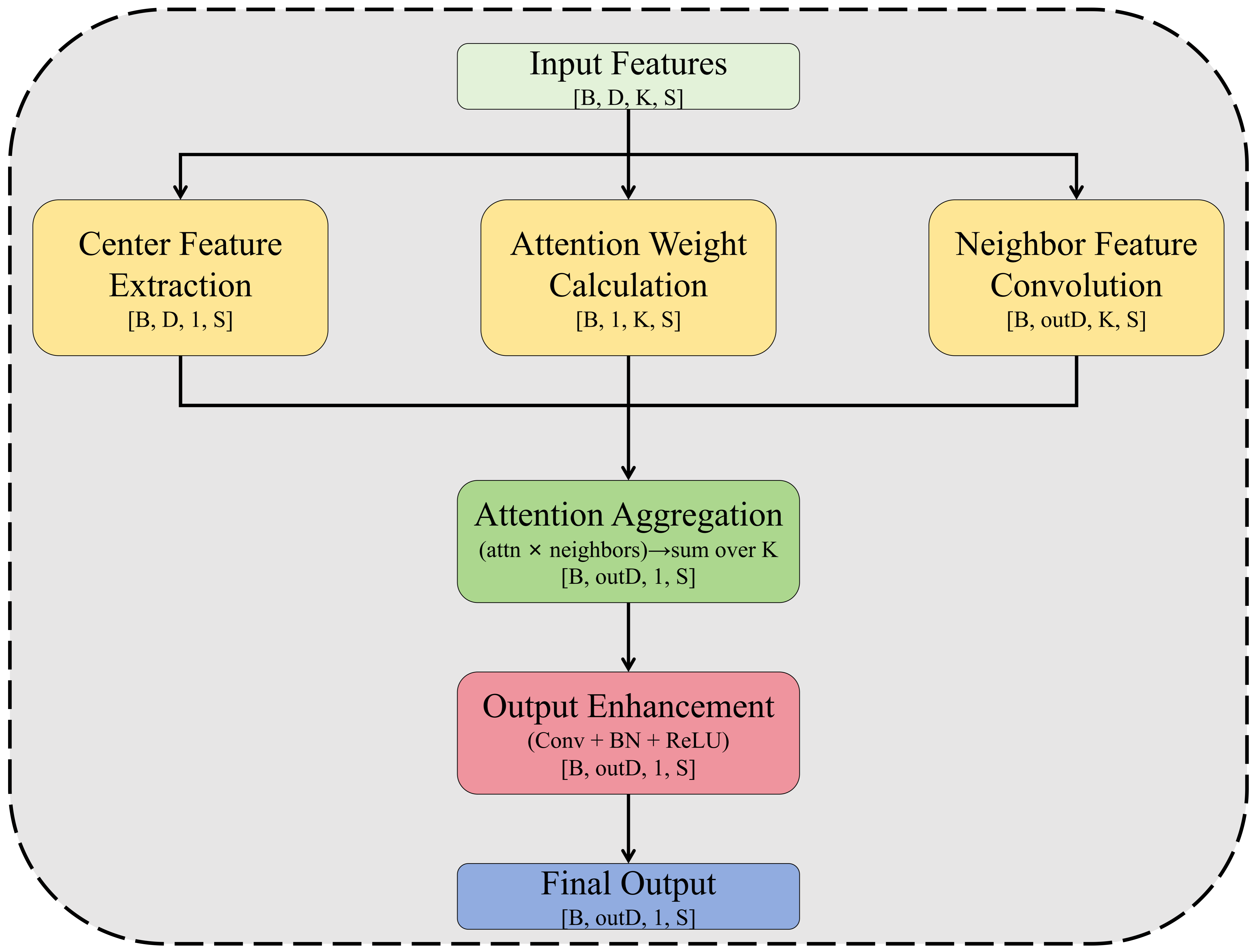

Supplement: Supplementary file 1 [file DataSheet1.zip › Figure/3.pdf]

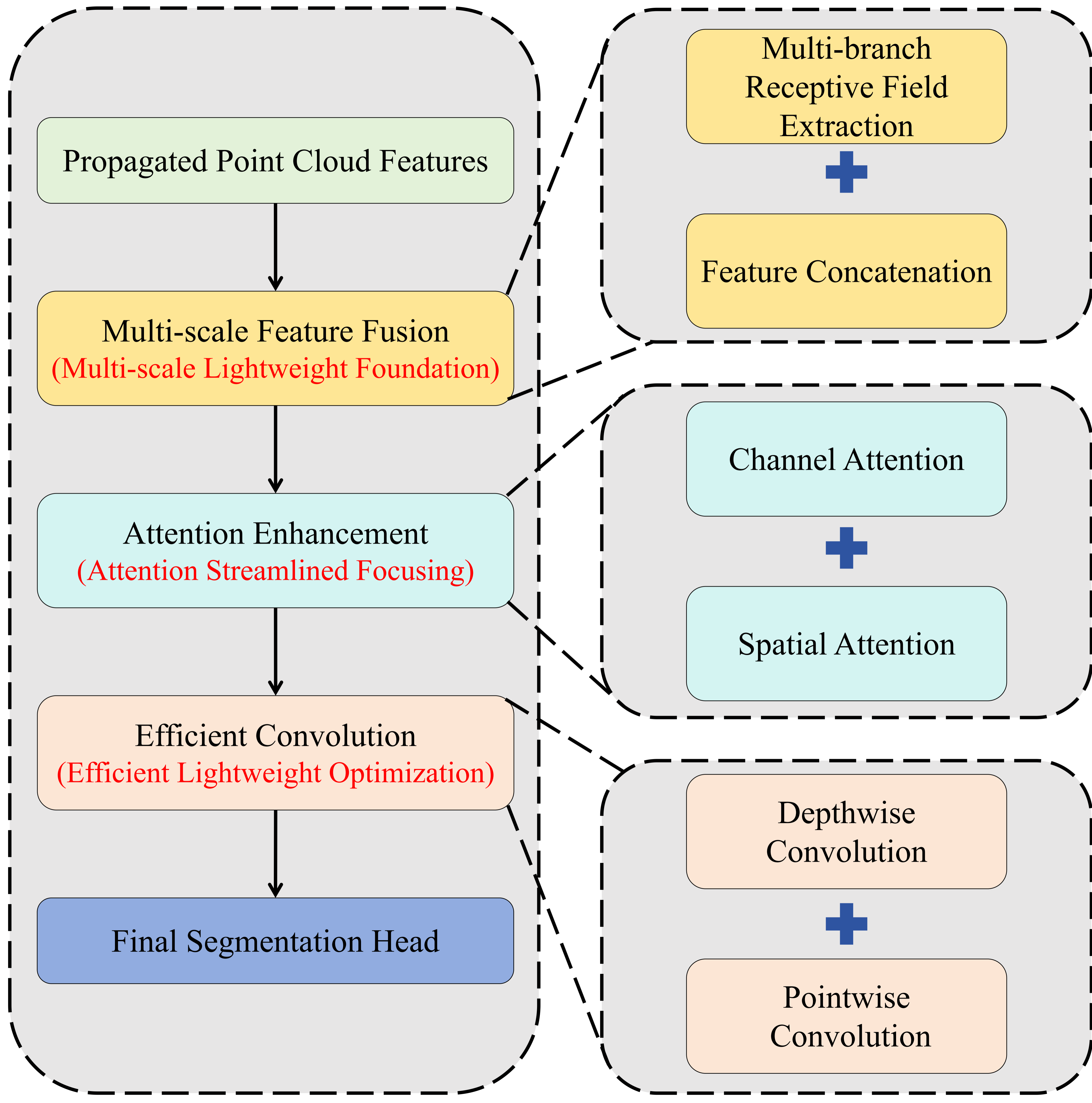

Supplement: Supplementary file 1 [file DataSheet1.zip › Figure/4.pdf]

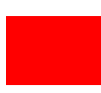 Cotton Foliage  
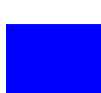 Cotton Ball

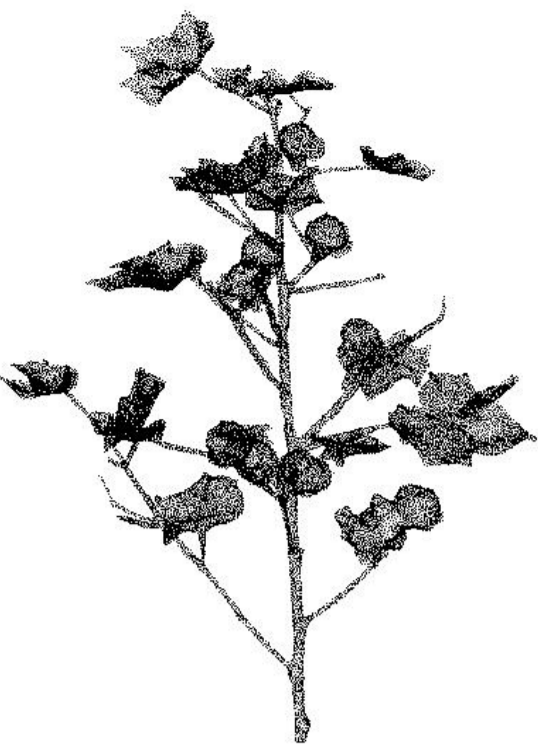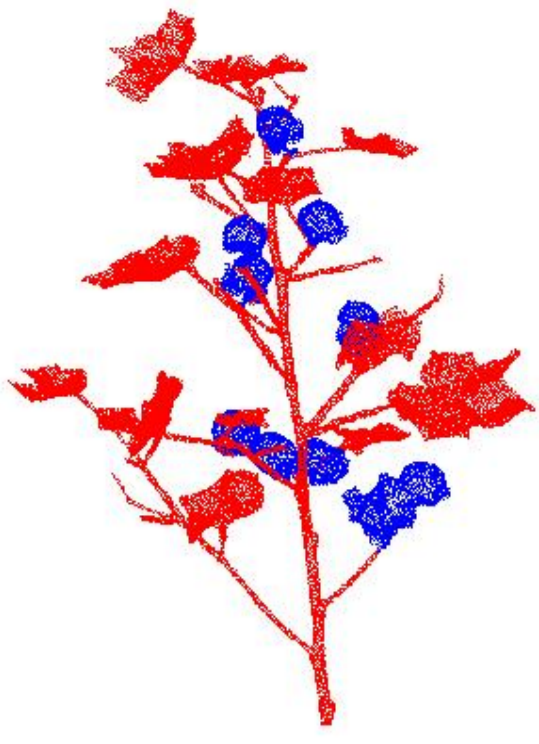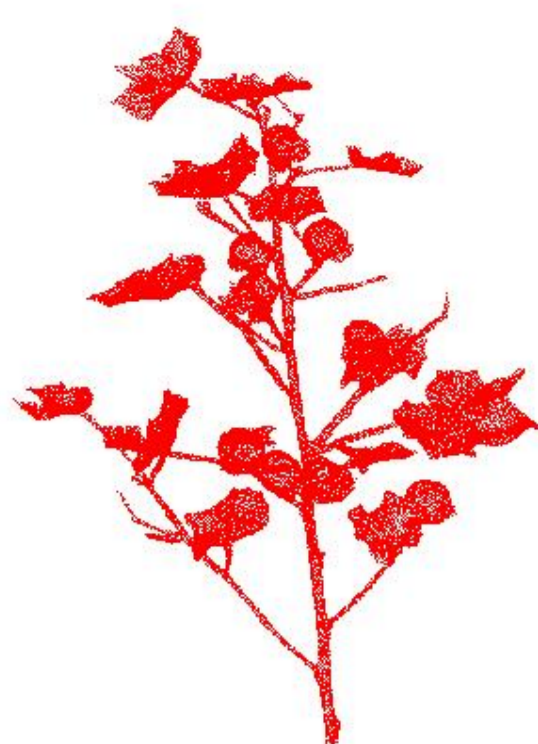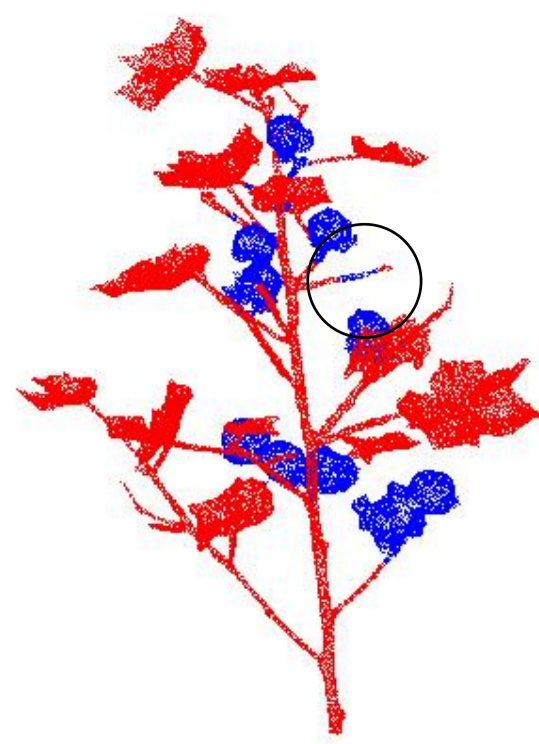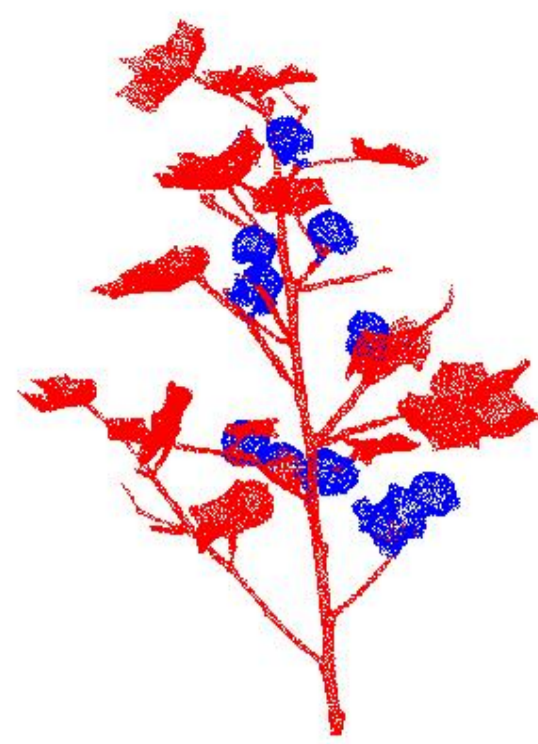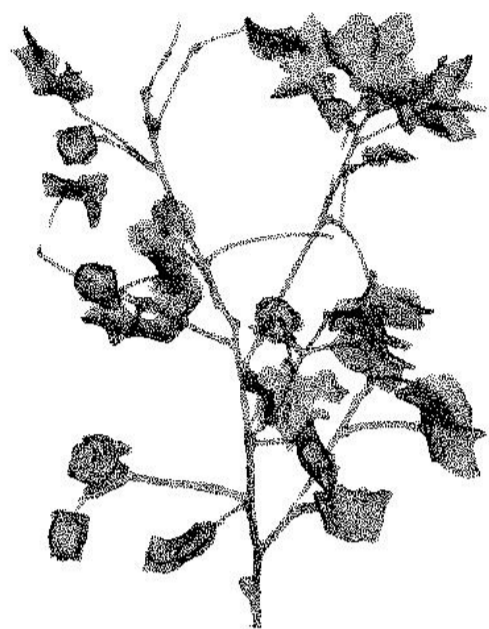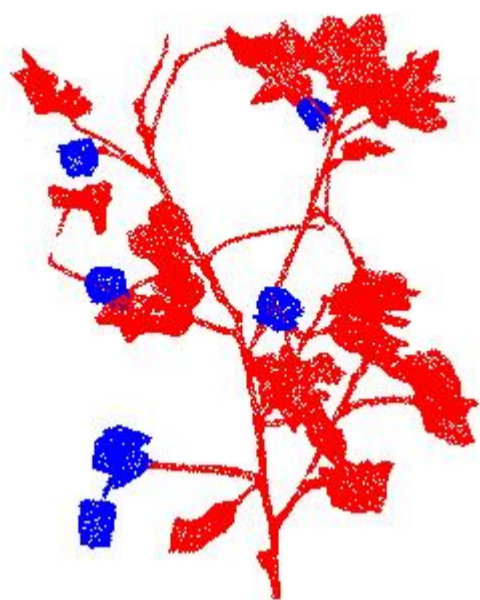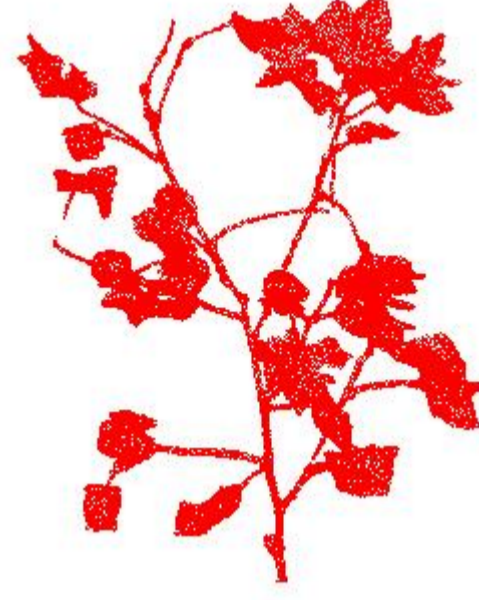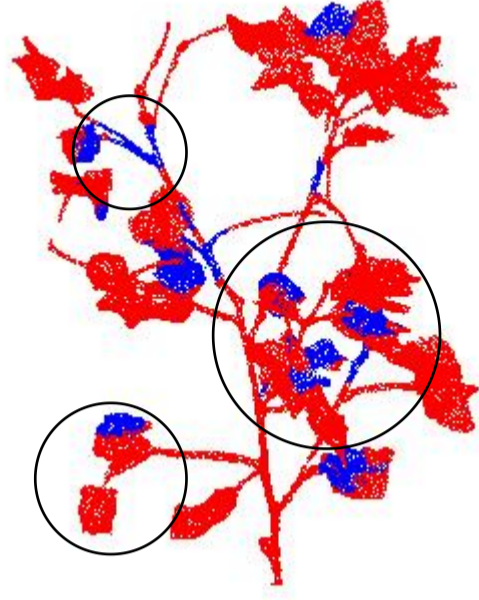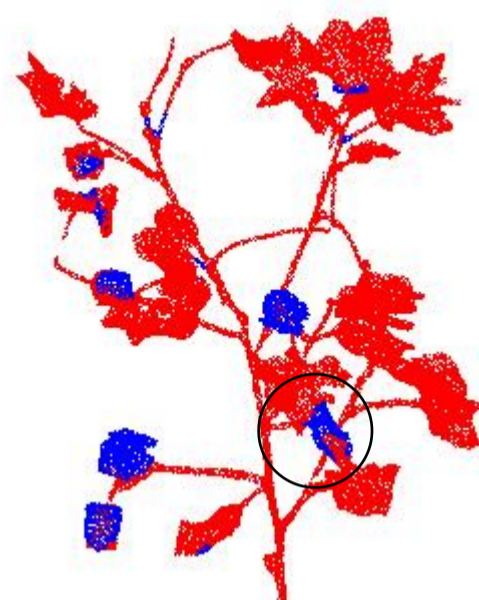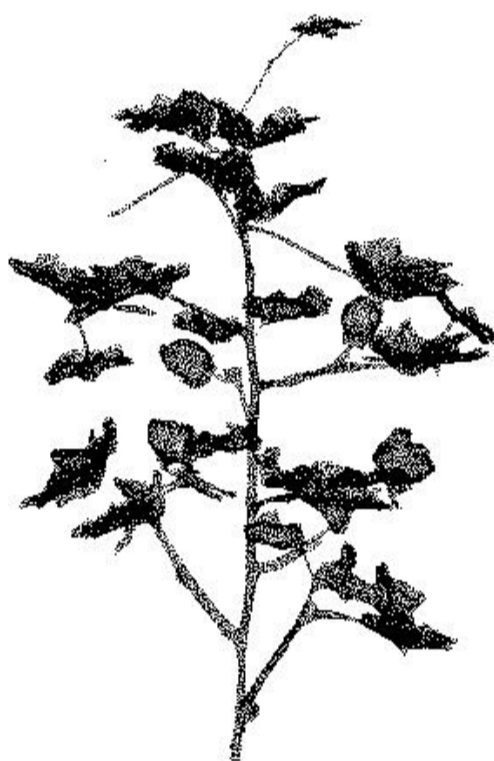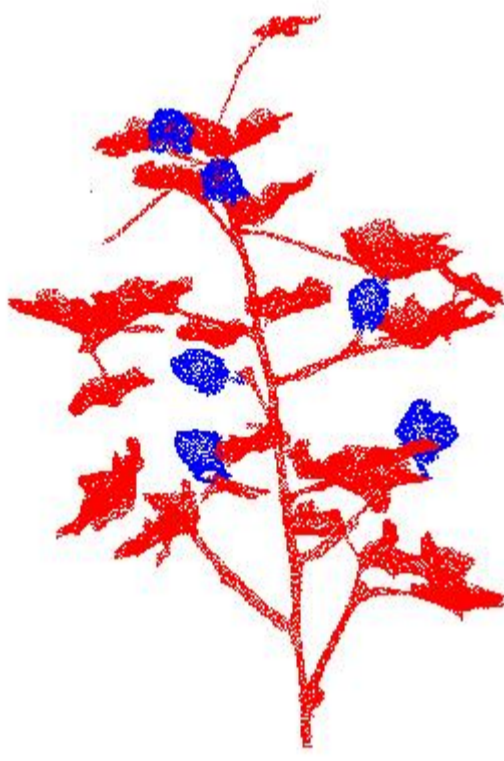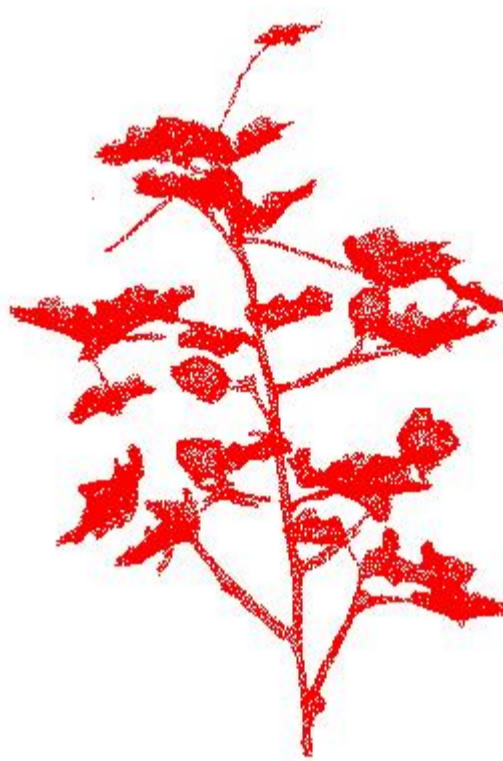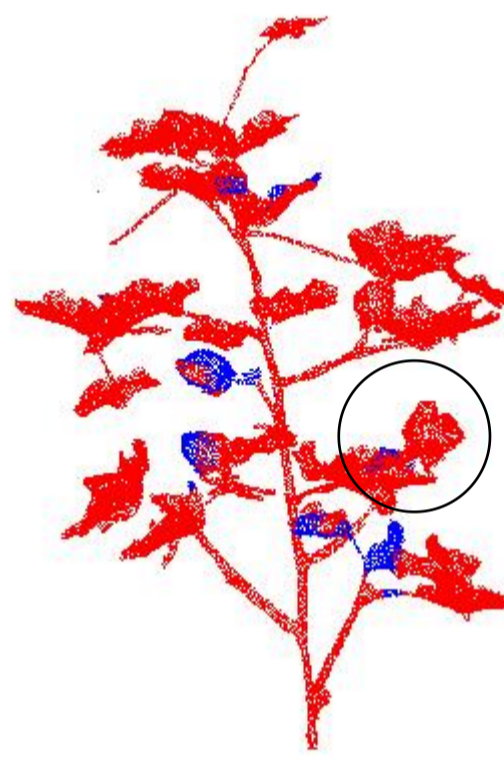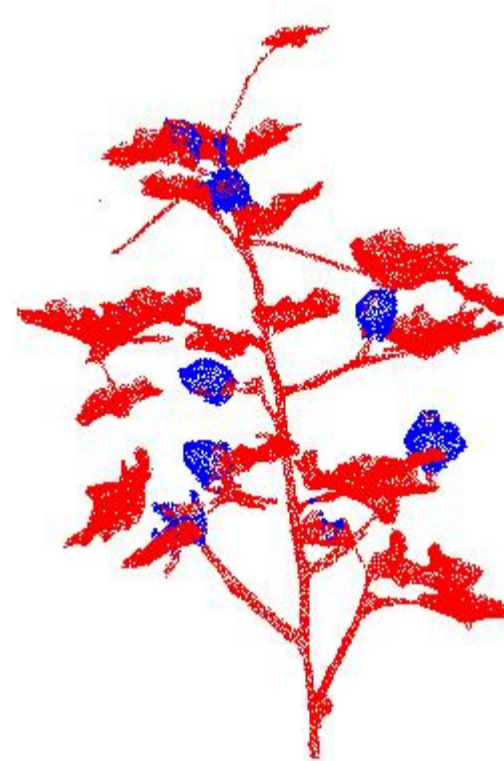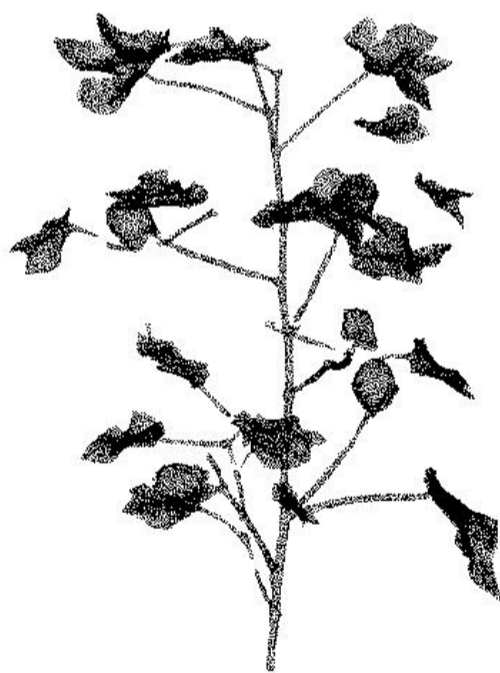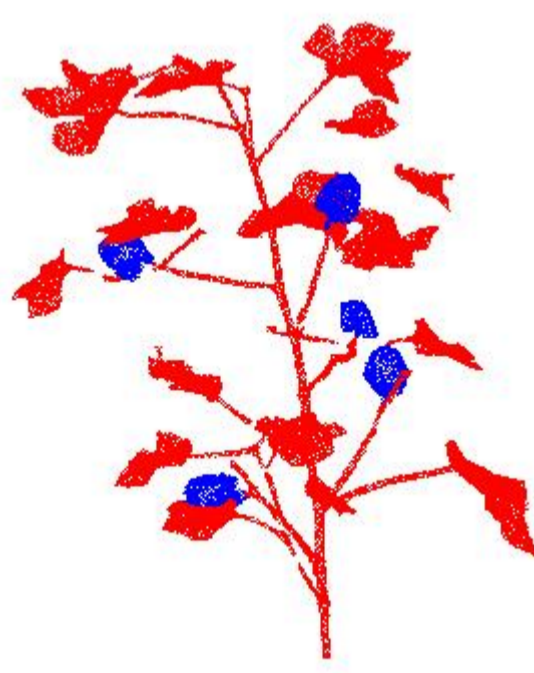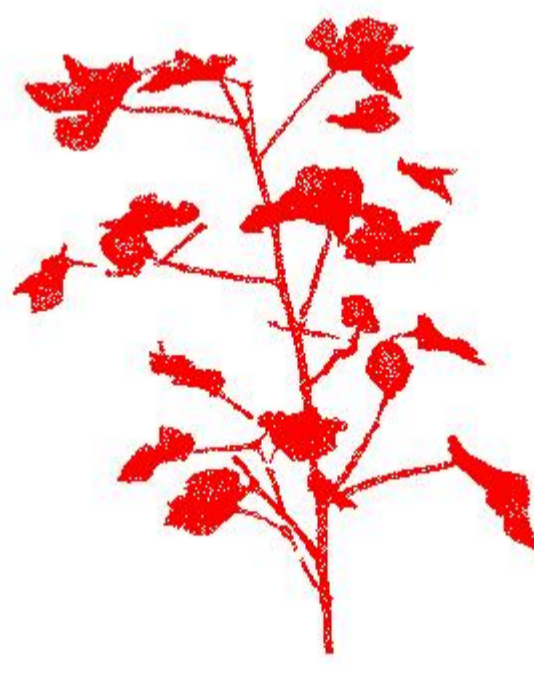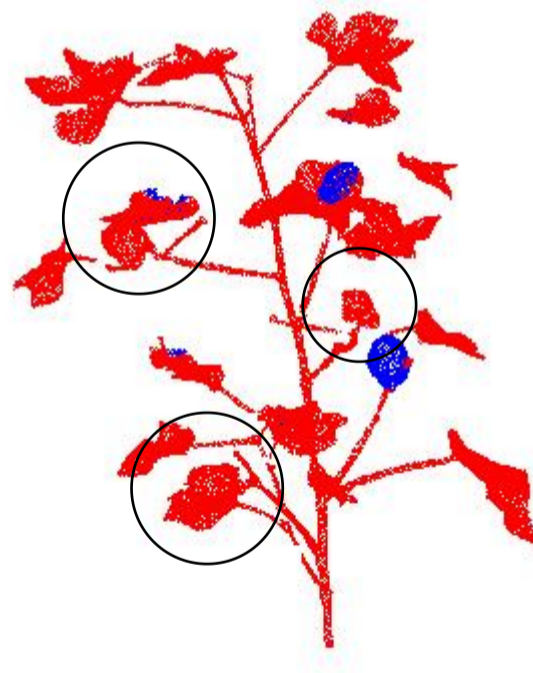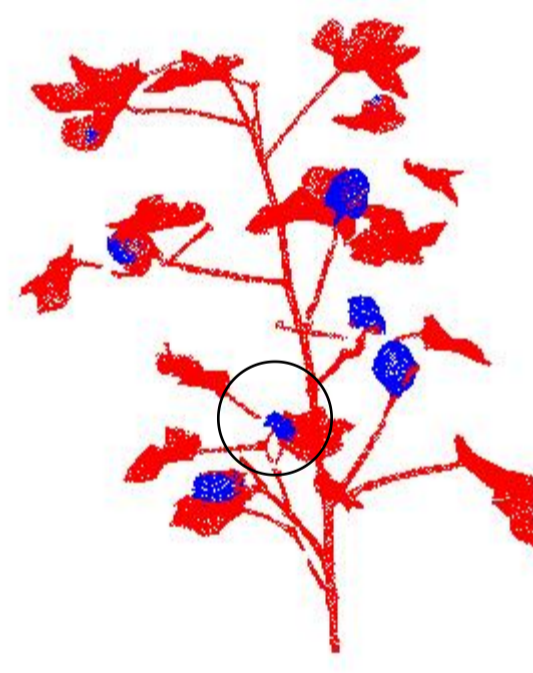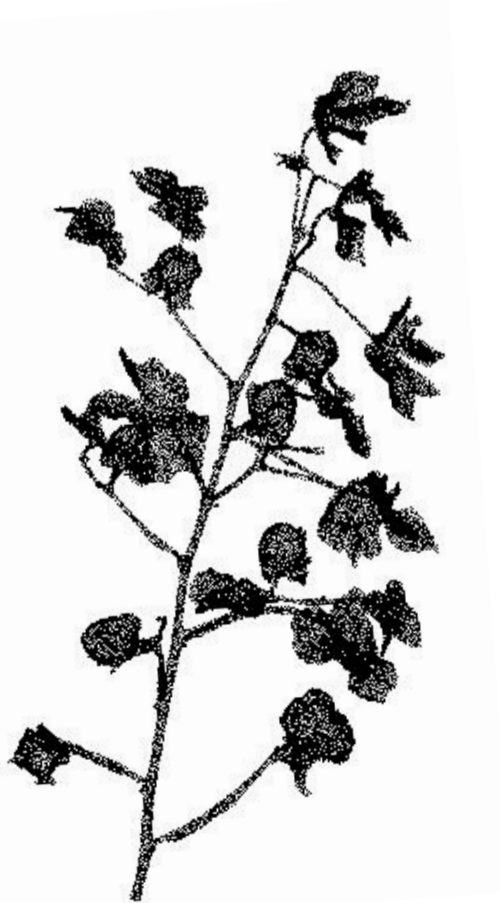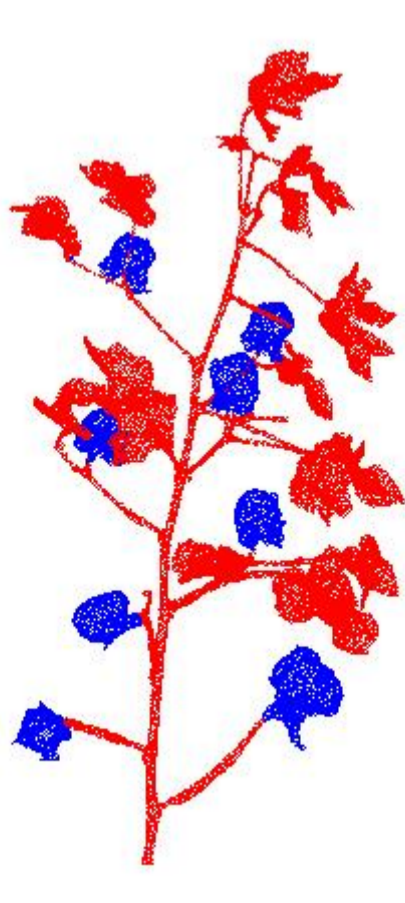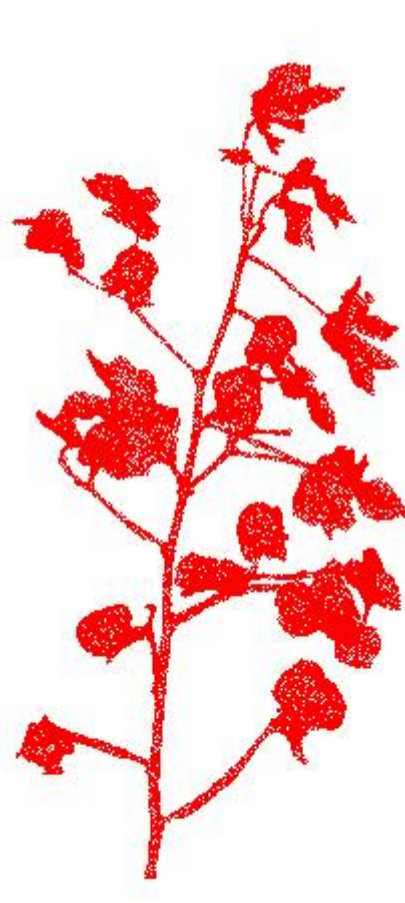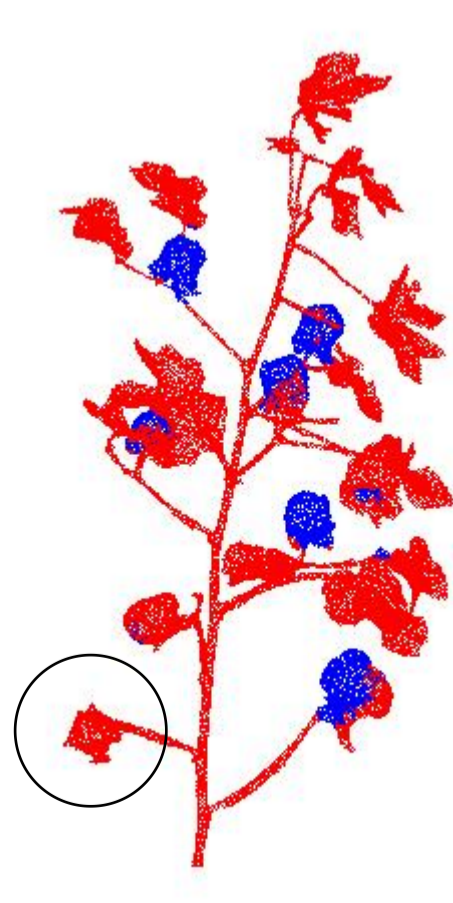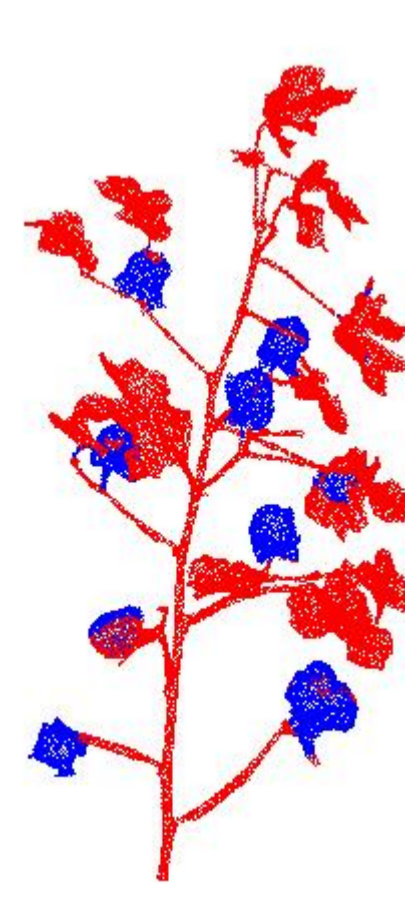

(A)

(B)

(C)

(D)

(E)

Supplement: Supplementary file 1 [file DataSheet1.zip › Figure/8.pdf]

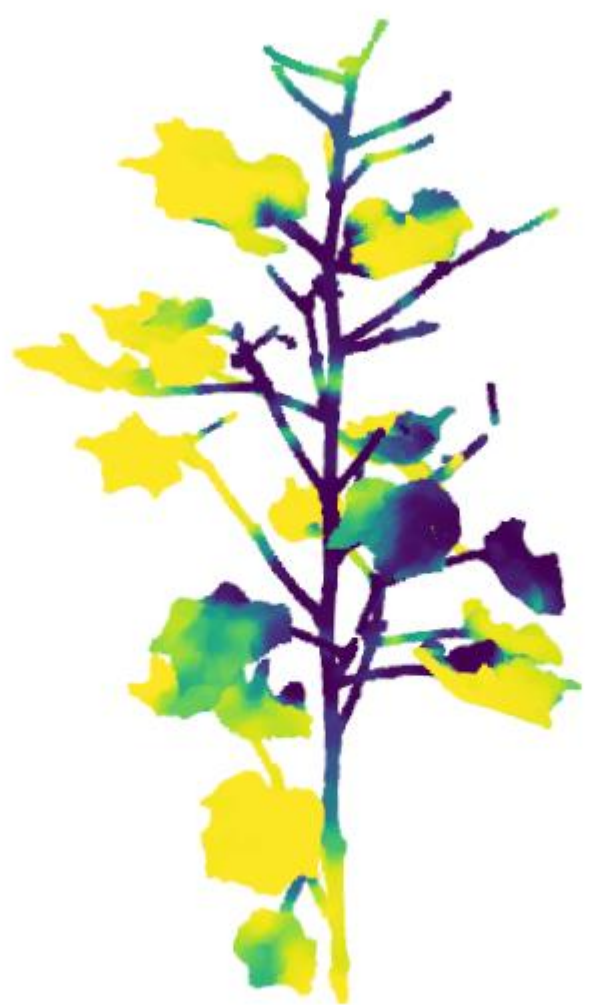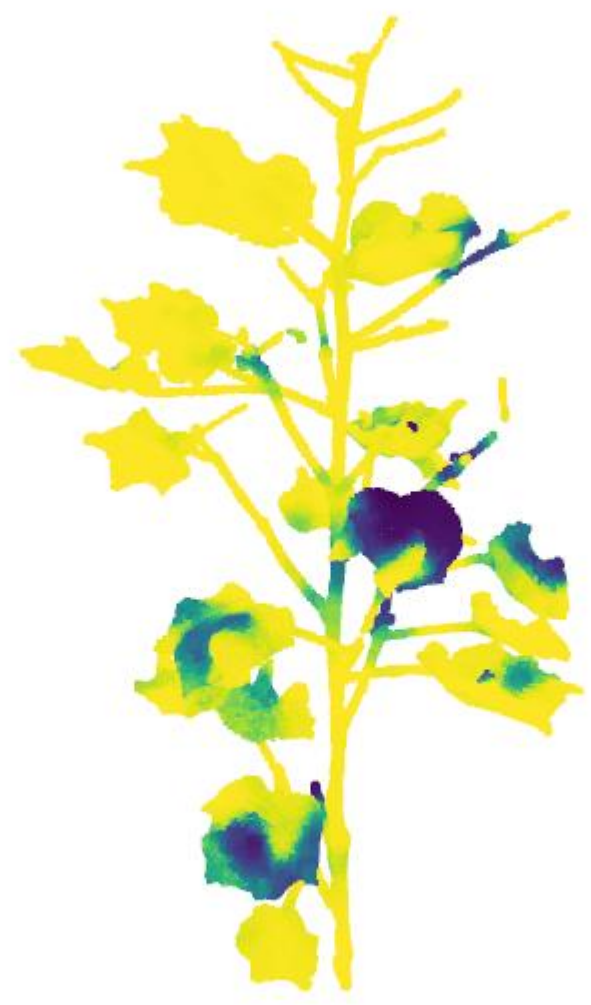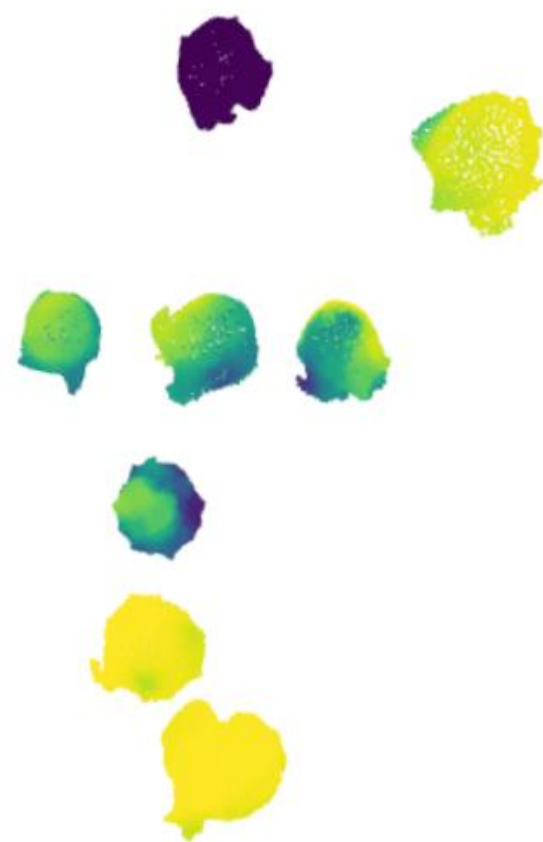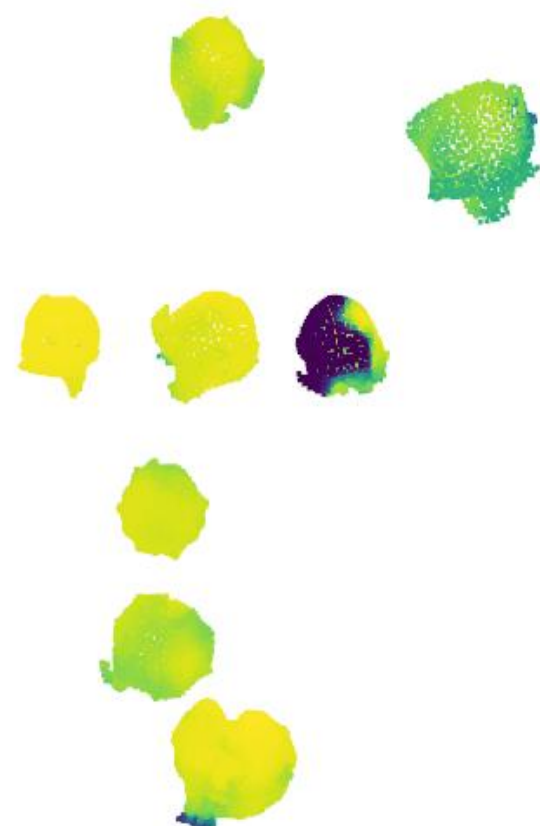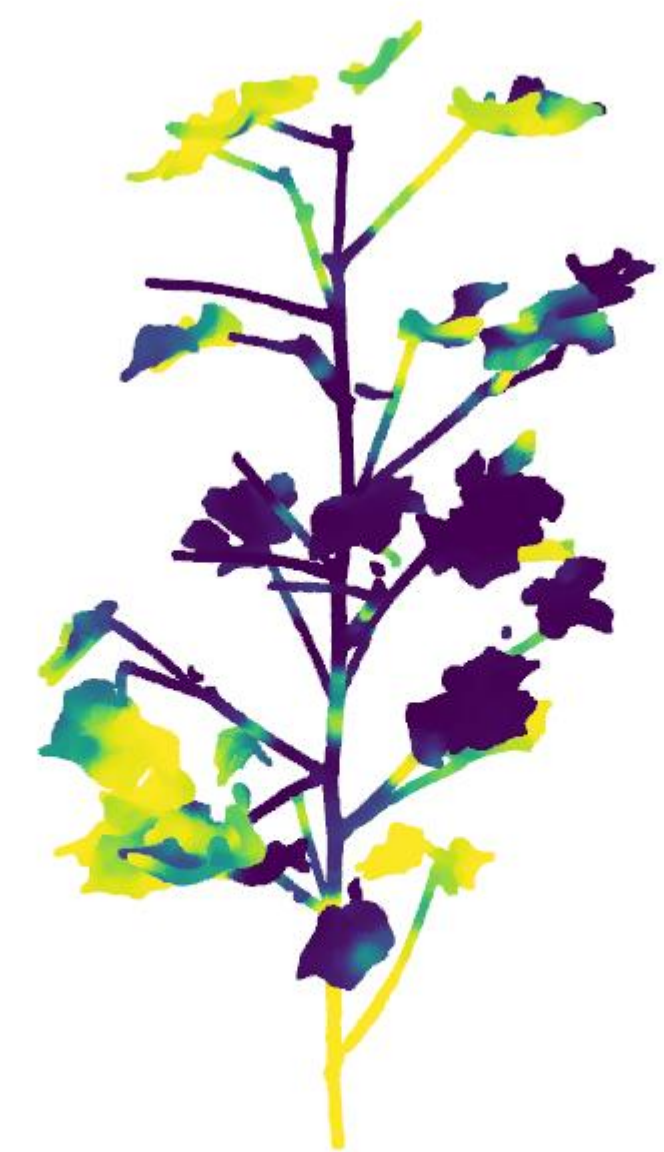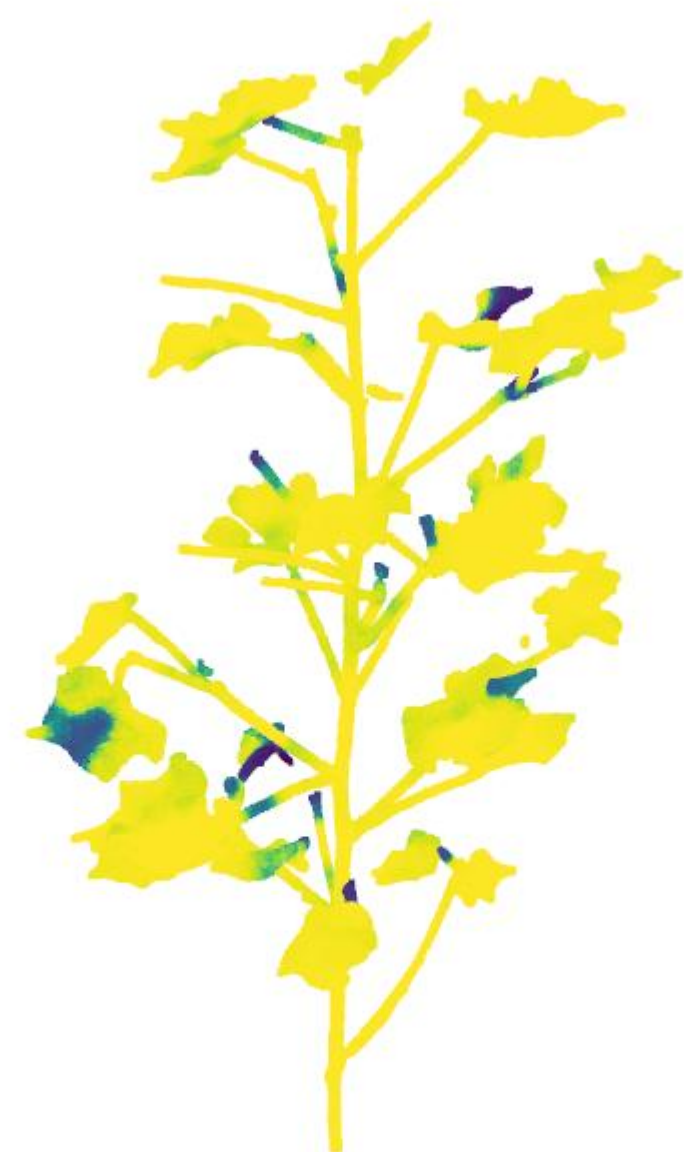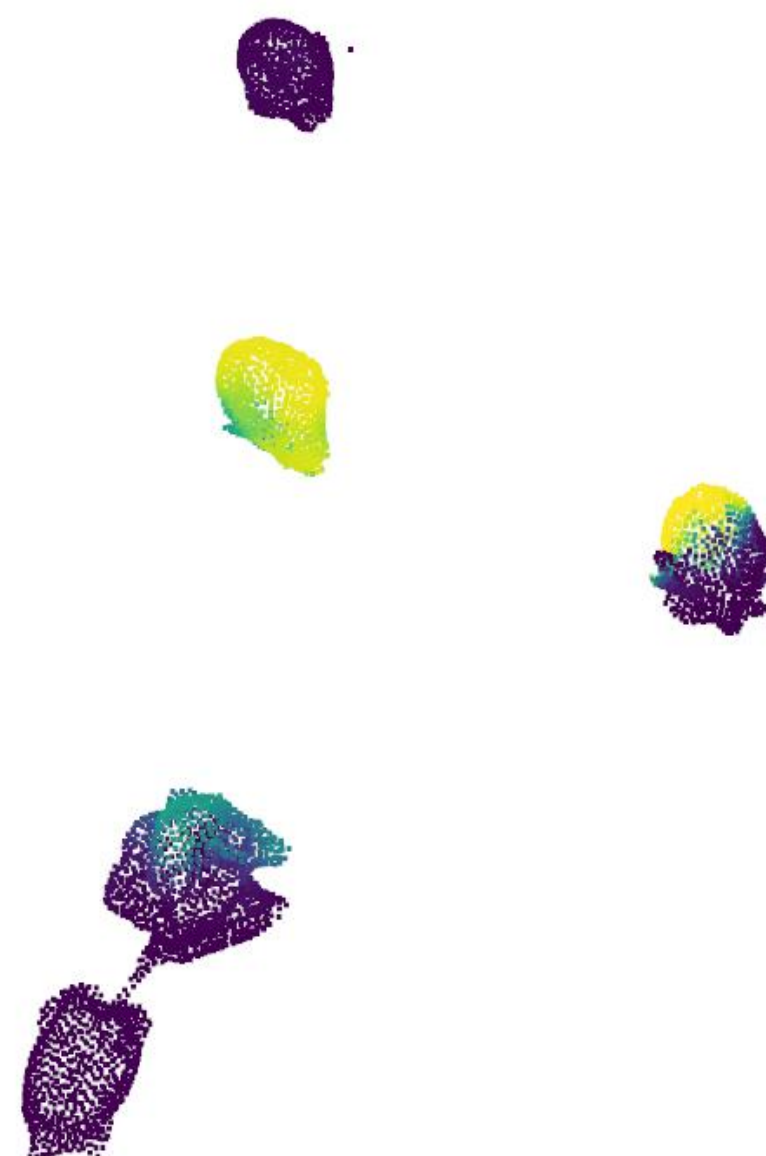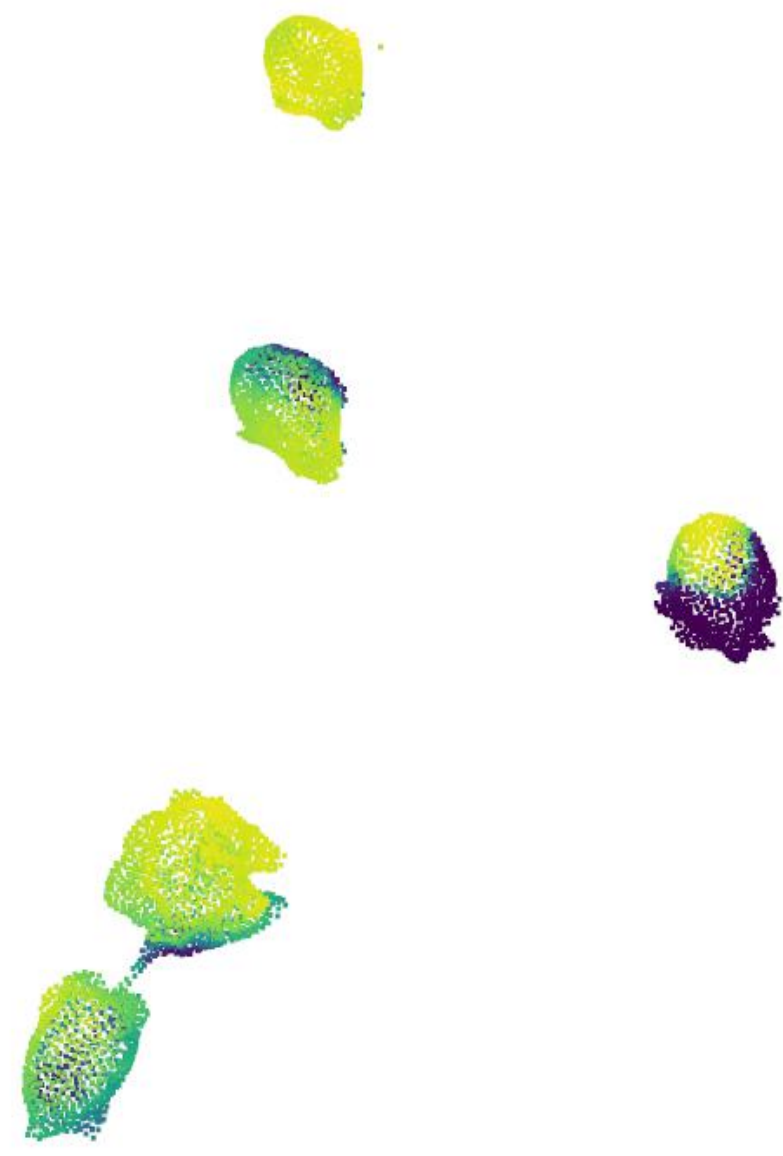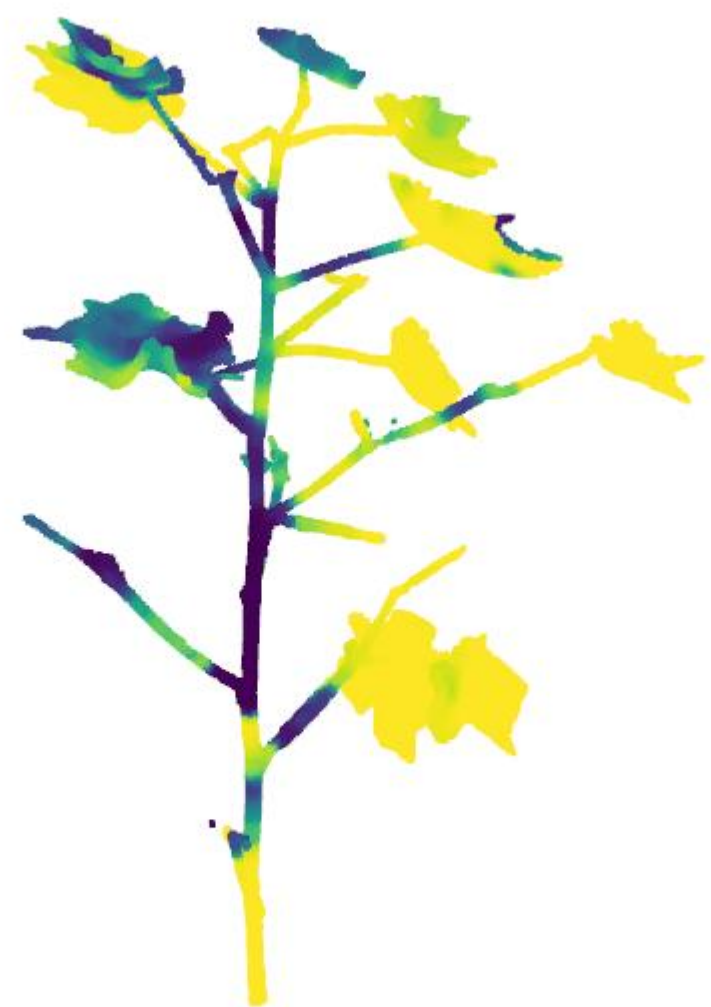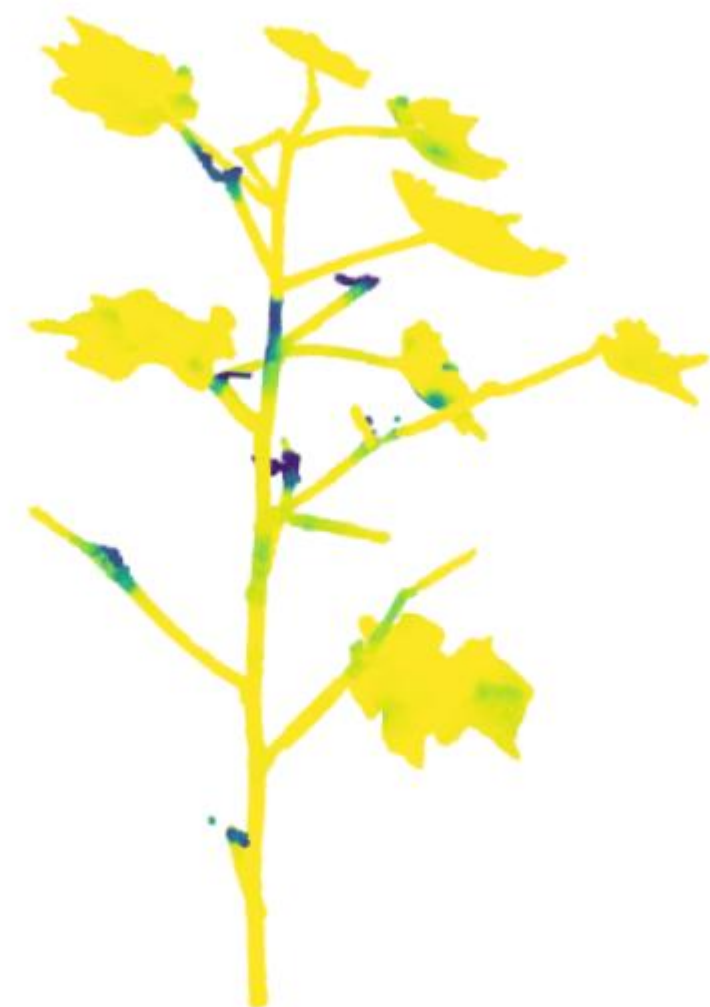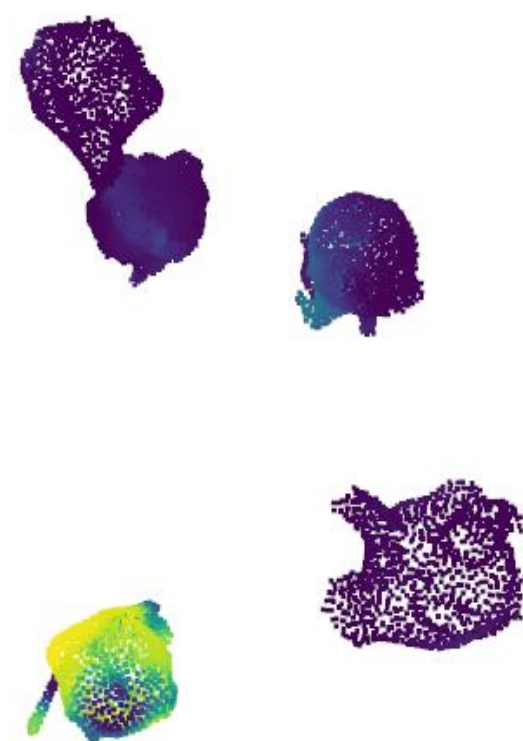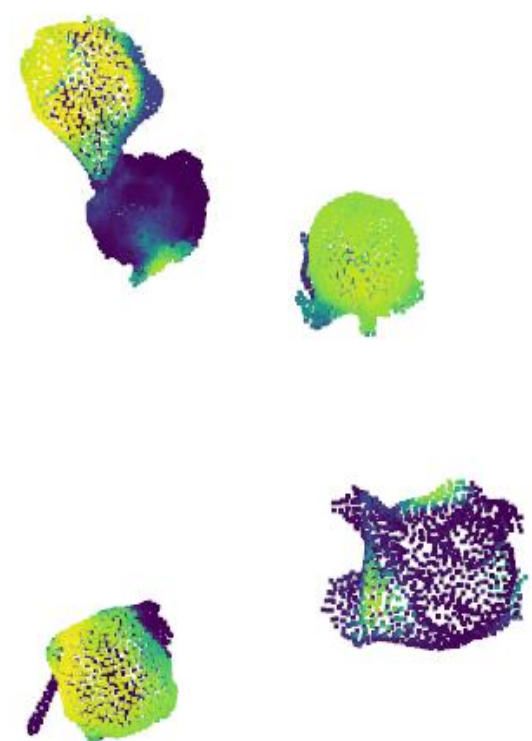

(A)

(B)

(C)

(D)

Supplement: Supplementary file 1 [file DataSheet1.zip › Figure/9.pdf]

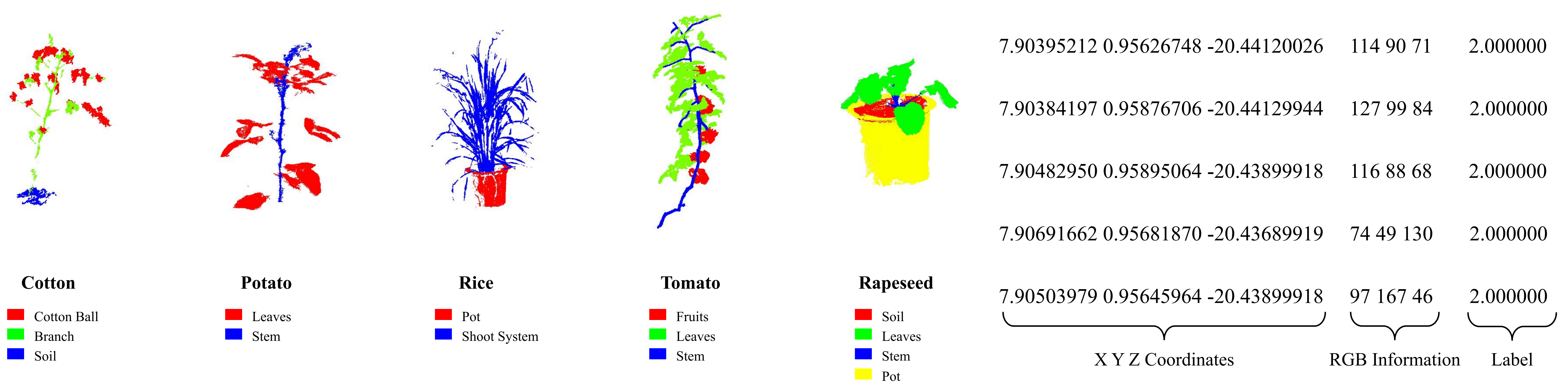

Supplement: Supplementary file 1 [file DataSheet1.zip › Figure/5.pdf]

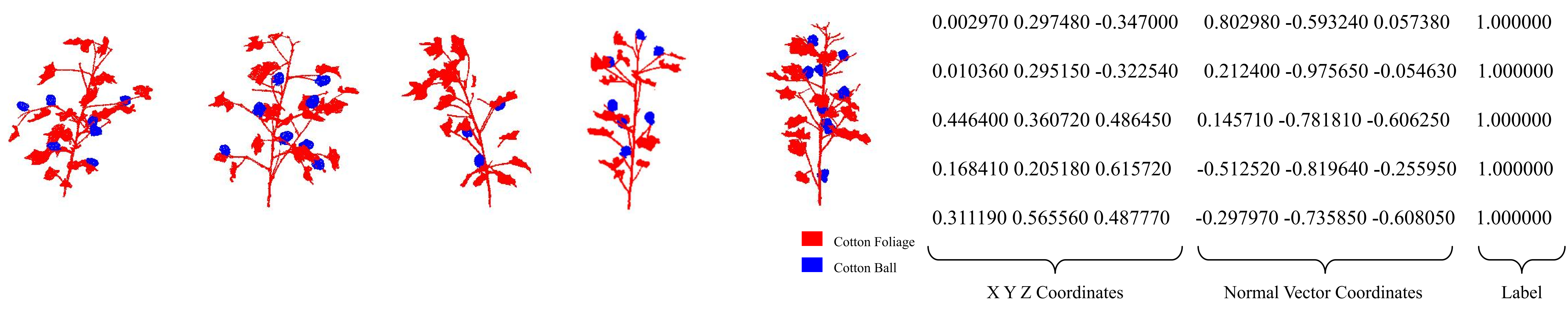

Supplement: Supplementary file 1 [file DataSheet1.zip › Figure/6.pdf]

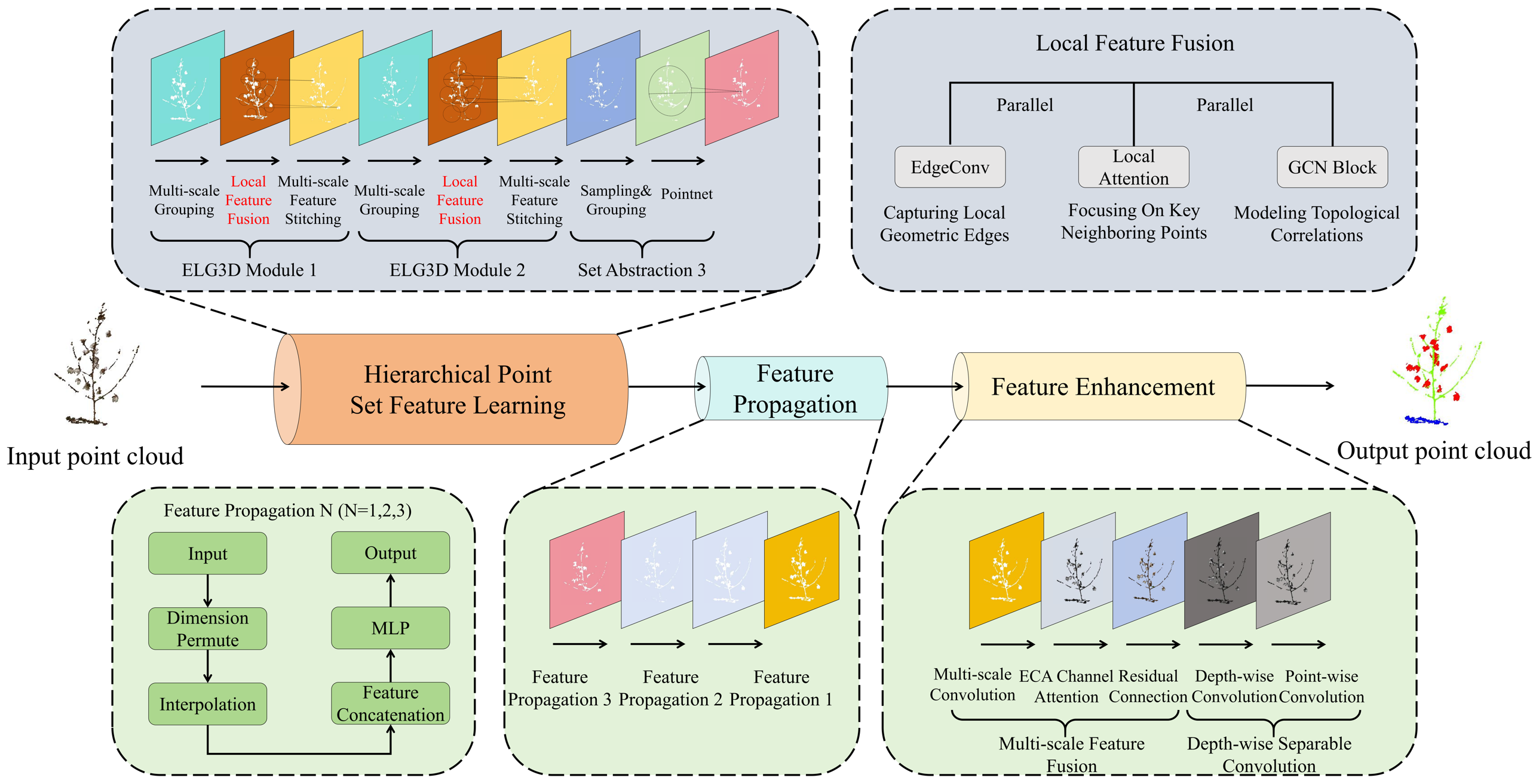

Supplement: Supplementary file 1 [file DataSheet1.zip › Figure/1.pdf]
